# Systems biology analysis of the *Clostridioides difficile* core-genome contextualizes microenvironmental evolutionary pressures leading to genotypic and phenotypic divergence

**DOI:** 10.1038/s41540-020-00151-9

**Published:** 2020-10-20

**Authors:** Charles J. Norsigian, Heather A. Danhof, Colleen K. Brand, Numan Oezguen, Firas S. Midani, Bernhard O. Palsson, Tor C. Savidge, Robert A. Britton, Jennifer K. Spinler, Jonathan M. Monk

**Affiliations:** 1 Department of Bioengineering, University of California, San Diego, La Jolla, CA USA; 2 Department of Molecular Virology and Microbiology, Baylor College of Medicine, Houston, TX USA; 3 Alkek Center for Metagenomics and Microbiome Research, Baylor College of Medicine, Houston, TX USA; 4 Department of Pathology and Immunology, Baylor College of Medicine, Houston, TX USA

**Keywords:** Structural biology, Systems analysis, Evolution

## Abstract

Hospital acquired *Clostridioides* (*Clostridium*) *difficile* infection is exacerbated by the continued evolution of *C. difficile* strains, a phenomenon studied by multiple laboratories using stock cultures specific to each laboratory. Intralaboratory evolution of strains contributes to interlaboratory variation in experimental results adding to the challenges of scientific rigor and reproducibility. To explore how microevolution of *C. difficile* within laboratories influences the metabolic capacity of an organism, three different laboratory stock isolates of the *C. difficile* 630 reference strain were whole-genome sequenced and profiled in over 180 nutrient environments using phenotypic microarrays. The results identified differences in growth dynamics for 32 carbon sources including trehalose, fructose, and mannose. An updated genome-scale model for *C. difficile* 630 was constructed and used to contextualize the 28 unique mutations observed between the stock cultures. The integration of phenotypic screens with model predictions identified pathways enabling catabolism of ethanolamine, salicin, arbutin, and N-acetyl-galactosamine that differentiated individual *C. difficile* 630 laboratory isolates. The reconstruction was used as a framework to analyze the core-genome of 415 publicly available *C. difficile* genomes and identify areas of metabolism prone to evolution within the species. Genes encoding enzymes and transporters involved in starch metabolism and iron acquisition were more variable while *C. difficile* distinct metabolic functions like Stickland fermentation were more consistent. A substitution in the trehalose PTS system was identified with potential implications in strain virulence. Thus, pairing genome-scale models with large-scale physiological and genomic data enables a mechanistic framework for studying the evolution of pathogens within microenvironments and will lead to predictive modeling to combat pathogen emergence.

## Introduction

*Clostridioides (Clostridium) difficile* continues to be a leading cause of hospital-borne infection, adversely affecting patient health as well as causing significant healthcare costs^1^. The continued evolution of *C. difficile* strains to both antibiotic resistance and survival in the host greatly increases the challenges of treatment^2^. *C. difficile* infection (CDI) occurs following the disruption of the host microbiota after treatment with antibiotics and instances of subsequent recurrent infections are common, often presenting with more severe symptoms^3^. In the absence of the natural microbiota, opportunistic, toxigenic strains of *C. difficile* flourish and produce enterotoxins resulting in the observed patient symptoms. These symptoms are wide-ranging and vary from completely asymptomatic to antibiotic-associated diarrhea to pseudomembranous colitis and even death. Frighteningly, the rate of success for commonly used antibiotics metronidazole and vancomycin is steadily falling^4^.

Studying this deadly pathogen in the laboratory requires well-characterized stock strains. Unfortunately, the evolution of stock cultures used in laboratory experiments has recently emerged as a major concern. This evolution can lead to the accumulation of genetic changes that have relevant physiological outcomes and may alter experimental results making it difficult to replicate results between labs. Recent studies identified seven mutations in commonly used stock strains of *Escherichia coli* K-12 MG1655 with implications for physiological experiments including loss of function of *glpR* and *crl*^5^. *C. difficile* is no exception to this phenomenon. Previous studies have demonstrated that accumulated mutations in stock strains can have physiological implications and even altered virulence in a hamster infection model^6^. Thus, with an explosion of research on *C. difficile* it is important to delineate mutations in stock strains and explain their physiological consequences.

To investigate the hypothesis that strains passaged in different laboratories would exhibit divergent phenotypes, we generated large-scale metabolic profiles of carbon utilization for three isolates of a reference strain commonly used in *C. difficile* research: CD630 isolates from two different laboratories as well as one close relative sensitive to the antibiotic erythromycin (CD630Δ*erm*). Furthermore, whole-genome sequencing of the strains allowed a comparison of both the genetic and phenotypic divergence amongst the three laboratory stock cultures. Genome-scale models (GEMs) of metabolism serve as a unifying platform to advance coordination of research and therapeutic advancements^7,8^. To contextualize the divergence in phenotype and genotype between our stock strains we built and used a new GEM of *C. difficile* 630.

GEMs offer a systems-level analysis of an organism’s metabolic capabilities and establish a formal relationship between genotype and phenotype^9^. Two previous reconstructions iMLTC806cdf^10^ and icdf834^11^ have been published for *C. difficile* strain 630. Here we present iCN900 that builds on iMLTC806cdf and icdf834, and reflects the most comprehensive knowledge base for *C. difficile* 630 to date. The model was used as a scaffold to interrogate the issue of stock culture evolution. We analyzed the core-genome of 415 strains to identify allelic sequence variants between genes determined to be present in each of the strains. Analyzing these genes within the metabolic network context provided by iCN900 illuminates which *C. difficile* metabolic pathways may be under evolutionary selective pressures. In addition, these data emphasize how laboratory-specific microenvironmental pressures on stock cultures contribute to divergent interlaboratory results that may hinder translational science limiting the development of new treatment options.

## Results

### High-throughput screens highlight phenotypic differences between three CD630 lab strains

**Fig. 1:**
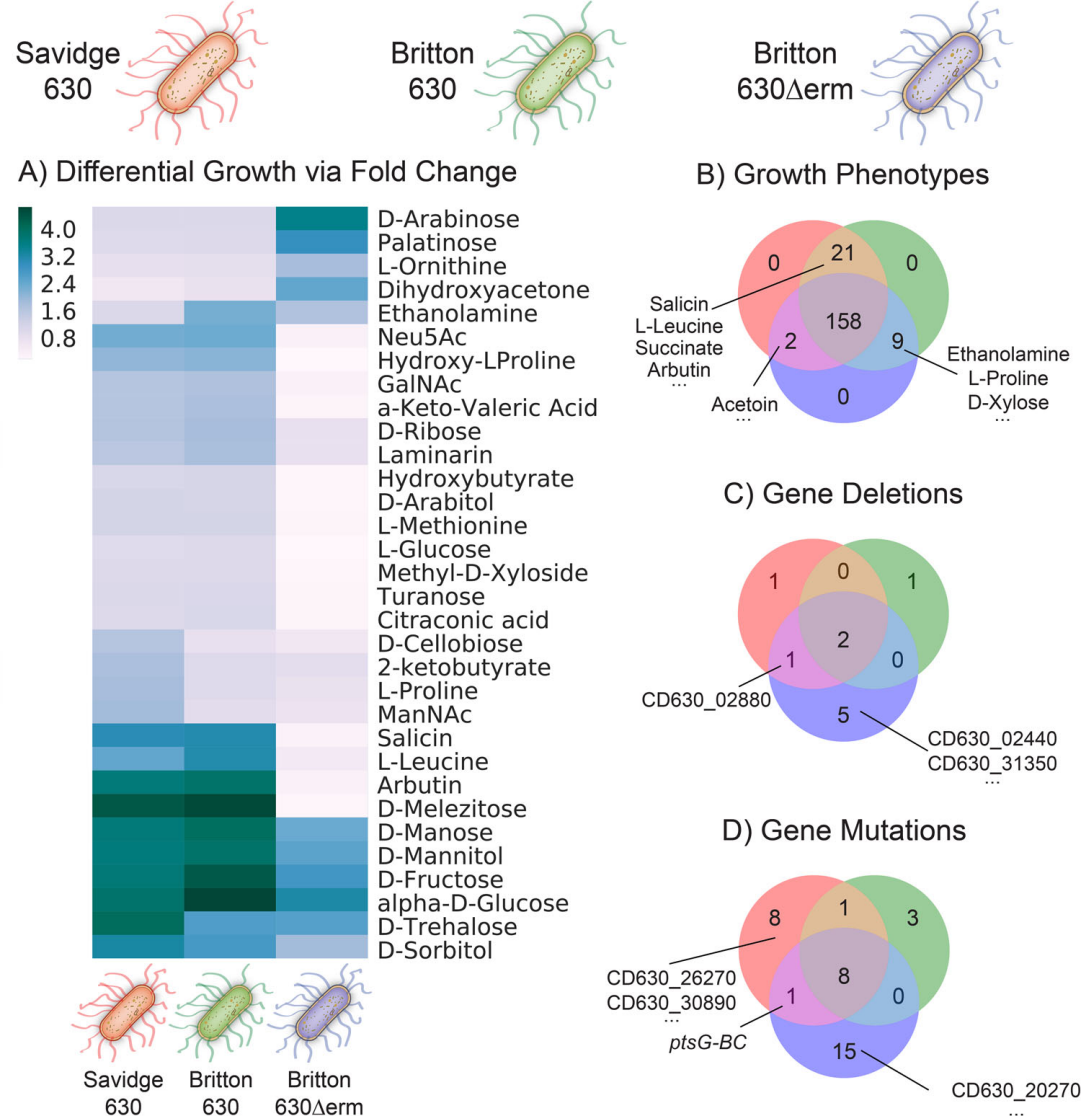
Experimental phenotyping of three different laboratory stock cultures of *C. difficile* 630. The Savidge 630, Britton 630, and Britton 630Δ*erm* are represented by red, green, and blue respectively. (**A**) Heat map of the maximal OD_620_ of *C. difficile* strains in Biolog phenotype microarray plates for which the fold change among the strains had the greatest standard deviation between the strains. Selected carbon substrates supporting differential fold change are shown. (*n* = 3 biological replicates per strain). (**B**) Venn diagram of 190 carbon substrates tested. All three strains shared 158 growth phenotypes, while 21 phenotypes were shared between Savidge 630 and Britton 630, 9 between Britton 630 and Britton 630Δ*erm*, and 2 phenotypes between Britton 630Δ*erm* and Savidge 630. (**C**) Venn diagram detailing the identified gene deletions of each strain versus the reference sequence. (**D**) Venn diagram detailing mutations of each strain versus the reference sequence.

To evaluate the phenotypic divergence of closely related strains, we selected three different laboratory strains of *C. difficile* 630 including the close relative knockout 630*Δerm* strain^6^ (Supplementary Table 1). We refer to the three strains as Savidge 630, Britton 630, and Britton 630Δ*erm* coinciding with their laboratory of origin, noting that the Britton 630 strain is not parental to Britton 630Δ*erm* (“Methods”). Phenotypic growth profiles of all three strains were generated in biological triplicate across 190 different carbon sources using Biolog Phenotype Microarrays^12^. Using the growth data generated from each *C. difficile* strain, we evaluated the phenotypic divergence of these closely related strains. Overall, each of the three laboratory *C. difficile* strains showed concordant phenotypes on 158 of the 190 compounds tested (Fig. 1B). Thirty-two (16.8%) compounds displayed varied growth phenotypes across this set of three lab-adapted CD630 strains including several notable differences that are interrogated using the GEM discussed below (Fig. 1A).

To robustly evaluate the genetic content of each of our three investigated laboratory 630 strains, we completed whole-genome sequencing and comparative genomics analyses to identify genetic differences relative to the reference 630 sequence (AM180355.1). We used breseq^13^ to identify single-nucleotide variants (SNVs) and gene deletions with respect to the reference sequence (Fig. 1C, D). Complete lists of predicted variants and deletions are available in Tables 1 and 2, respectively (Supplementary Data File 1). Seven variants previously noted as likely mistakes in the original *C. difficile* 630 AM180355.1 reference assembly^6^ were identified in all three strains. An additional synonymous SNV (E304E (GAG → GAA)) within the aminotransferase gene CD630_25320 was identified as common in all three strains. The Savidge 630, Britton 630, and Britton 630Δ*erm* each had 8, 3, and 15 unique SNVs relative to the reference (Table 1).

**Table 1:** Comparison of SNVs detected across three *C. difficile* 630 laboratory stock strains.

Gene	Description	Mutation	Annotation	Savidge 630	Britton 630	Britton 630Δerm
CD630_05730/thrS	Putative membrane protein/Threonyl-tRNA synthetase	C → T	Intergenic (+173/−843)	✓	✓	✓
CD630_05770/CD630_05780	Conserved hypothetical protein/transporter, Major Facilitator superfamily (MFS)	A → T	Intergenic (−126/+35)	✓	✓	✓
CD630_24550/CD630_24560	Putative CRISPR-associated protein/ABC-type transport system, sugar-family ATP-binding protein	G → T	Intergenic(−543/−193)	✓	✓	✓
rplC	50S ribosomal protein L3	G → T	G114G (GGG → GGT)	✓	✓	✓
CD630_11900	Putative acetyltransferase	T → C	F133L (TTT → CTT)	✓	✓	✓
CD630_17670	Glyceraldehyde-3-phosphate dehydrogenase (GAPDH)	C → G	P33A (CCC → GCC)	✓	✓	✓
CD630_25320	Aminotransferase, alanine-glyoxylate transaminase	C → T	E304E (GAG → GAA)	✓	✓	✓
CD630_13880	Putative transcriptional regulator	(T) _6→7_	Coding (40/45 nt)	✓	✓	✓
CD630_31561	Fragment of conserved hypothetical protein	+A	Coding (309/339 nt)	✓	–	✓
CD630_34170/CD630_34180	ABC-type transport system, sugar-family ATP-binding protein/Precorrin-2 dehydrogenase	A → G	Intergenic (−3769/+1786)	✓	✓	–
CD630_34170/CD630_34180	ABC-type transport system, sugar-family ATP-binding protein/Precorrin-2 dehydrogenase	+ C	Intergenic(−3628/+1927)	✓	–	–
CD630_19000/CD630_19010	conserved hypothetical protein/Transcriptional regulator, Phage-type	A → T	Intergenic (−160/−294)	✓	–	–
CD630_02050	Transcription antiterminator, PTS operon regulator	G → T	G165C (GGT → TGT)	✓	–	–
CD630_26850	Putative sporulation stage II, protein E	Δ21 bp	Coding (339–359/1770 nt)	✓	–	–
CD630_32450	Transcriptional regulator, sigma-54 dependent	C → T	E261K (GAA → AAA)	✓	–	–
CD630_26270	Conserved hypothetical protein	C → A	G68C (GGT → TGT)	✓	–	–
CD630_30890	PTS system, glucose-like IIBC component	T → G	E258D (GAA → GAC)	✓	–	–
CD630_26670	PTS system, glucose specific IIBC component	C → T	V228I (GTT → ATT)	✓	–	–
CD630_26670	PTS system, glucose specific IIBC component	A → C	*524E (TAA → GAA)	–	–	✓
CD630_26670	PTS system, glucose specific IIBC component	(T) _8→7_	Coding (1558/1572 nt)	–	–	✓
CD630_20270	N-carbamoyl-L-amino acid hydrolase	G → A	G373E (GGG → GAG)	–	–	✓
CD630_06430	Two-component response regulator	T → C	I199I (ATT → ATC)	–	–	✓
CD630_07610	Putative ATP-dependent RNA helicase	G → T	D136Y (GAC → TAC)	–	–	✓
CD630_12480	ribonuclese III	G → T	G59V (GGC → GTC)	–	–	✓
CD630_14040	Putative oligopeptide transporter	A → G	E536G (GAA → GGA)	–	–	✓
CD630_12740	DNA topoisomerase I	C → T	Q386* (CAA → TAA)	–	–	✓
CD630_22630	Peptidyl-prolyl cis-trans isomerase, PpiC-type	G → T	S127* (TCA → TAA)	–	–	✓
CD630_22670	Fragment of membrane protein, abortive infection-type protein	(A) _5→6_	Coding (280/321 nt)	–	–	✓
CD630_29430	Putative phage replication protein	T → C	N210D (AAT → GAT)	–	–	✓
CD630_33790	Putative conjugate transposon protein Tn916-like, CTn7-Orf15	C → A	E63D (GAG → GAT)	–	–	✓
CD630_33980	Putative hydrolase, NUDIX family	C → A	G9C (GGT → TGT)	–	–	✓
CD630_30360/CD630_30370	Transporter, Major Facilitator Superfamily (MFS)/Transcriptional regulator, CarD family	G → T	Intergenic (−1521/+386)	–	–	✓
treR	Transcriptional regulator, GntR family	Δ6 bp	coding (192–197/723 nt)	–	–	✓
CD630_12060	Putative membrane protein	A → T	K120N (AAA → AAT)	–	✓	–
CD630_27920	Protein translocase subunit secA 2	T → A	P669P (CCA → CCT)	–	✓	–
CD630_31840/CD630_31850	Diaminopropionate ammonia-lysase/Fragment of putative RNA-binding protein	Δ9 bp	Intergenic (−393/+222)	–	✓	–

**Table 2:** Comparison of deletions detected across three *C. difficile* 630 laboratory stock strains.

Gene	Description	Savidge 630	Britton 630	Britton 630Δerm
CD630_10250	ABC-type transport system, spermidine/putrescine permease	1197421:1197442	1197421:1197445	1197421:1197443
CD630_34170/CD630_34180	ABC-type transport system, sugar-family ATP-binding protein/Precorrin-2 dehydrogenase	4004552–4006944 :4007449–4007360	4004579–4006943 :4007462–4007423	4004577–4006952 :4007434–4007372
CD630_02880	PTS system, mannose/fructose/sorbose IIC component	348613:348633	–	348613:348633
CD630_01960	Fragment of conserved hypothetical protein, DUF111 family	255270:255292	–	–
CD630_12100	conserved hypothetical protein	–	1409337–1409360	–
CD630_02440/CD630_02450	Putative CDP-glecerol:Poly(glycerophosphate) glycerophosphotransferase/Flagellar basal-body rod protein FlgB	–	–	309199–309207
CD630_31350	Putative fructose-1-6-biphosphate adolase	–	–	3654239–3654260
CD630_09390-[CD630_09770]	46 genes: putative phage protein	–	–	1110626–1125088 :1141390–1125187
[CD630_28900]-CD630_29521	44 genes: putative phage protein	–	–	3381246–3397454 :3412003–3397553
[CD630_20060]-[ermB1]	8 genes	–	–	2316723–2317718 :2320011–2319025

Two gene deletions were identified common to all three genomes and a third deletion was present only in 630 Savidge and Britton 630Δ*erm*. Savidge 630 and Britton 630 each contained a single independent deletion relative to the reference in conserved hypothetical proteins CD630_01960 and CD630_12100, respectively. In contrast, Britton 630Δ*erm* contained five unique deletions not present in Savidge or Britton 630 (See Table 2). Three of the deletions unique to Britton 630Δ*erm* are two groups of 44 and 46 genes annotated as putative phage genes and the expected 8 genes loss for the erythromycin-sensitive derivative. In order to contextualize the remaining genetic differences distinguishing these three strains from each other and their impact on the observed phenotypic divergence we updated and deployed a GEM of *C. difficile* 630 metabolism.

### Genome-scale network reconstructions contextualize genetic divergence by serving as a scaffold for structural systems biology analysis

GEMs offer a powerful tool to contextualize and explain the effect of genetic changes in pathogenic organisms that impact human health. Therefore, we evaluated and updated a genome-scale network reconstruction of *C. difficile* 630 (Supplementary Text 1). The new *C. difficile* GEM, iCN900, contains an additional 66 genes, 46 reactions, and 70 metabolites compared to previous models of this strain (Supplementary Table 2). New content was incorporated into the reconstruction using both bioinformatic tools and manual curation. We implemented several tools to add new content to the reconstructions including the enzyme detection tool DETECT v2^14^, searching for homologs in closely related reconstructions^15^, and manual curation of pathways based on false negative model predictions against experimental data (“Methods”). This allowed for the inclusion of new transport reactions as well as significant refinement of the accuracy of the gene product rules for existing transporters. GEM additions included reactions for tRNA synthetase, carbon and sulfur metabolism, and cell envelope biosynthesis.

In addition to adding new content to the genome-scale network reconstruction for *C. difficile* 630, another major area of improvement was the removal of erroneous energy generating cycles (EGCs). EGCs allow for free energy generation during flux balance analysis (FBA) simulations and have been shown to be a prevalent problem in many non-curated GEM predictions. We implemented an existing algorithm^16,17^ to identify and confirm the existence of EGCs in the previous model (icdf834). We manually investigated icdf834 and found erroneous EGCs for ten energy carrying metabolites. We edited the reversibility of 29 reactions (Supplementary Table 3) to remedy these cycles making the network completely devoid of EGCs and therefore better suited to make accurate flux predictions utilizing FBA^18^. For a complete list of the EGCs originally present as well as the corresponding changes made to correct them see Supplementary Table 3. Finally, we updated the model nomenclature to align it with the BiGG standard, making its contents directly comparable with over 100 reconstructions of diverse organisms present in the BiGG database^19,20^. This improved model, iCN900, is available in the BiGG database and as Supplementary Data Files 2–4.

Recent studies have supplemented GEMs with protein structures to form GEM-PROs resulting in expanded applications for both genome- and protein-scale models^21,22^. This approach has enabled further contextualization of SNVs within the metabolic network. Protein structures have never been incorporated with a GEM of *C. difficile*, therefore we evaluated the current state of structural data available for *C. difficile* by mapping protein structures to the Protein Data Bank (PDB). Overall 1221 genes within the 630 reference genome map to a structure within the PDB. A subset of 524 of these genes are contained within iCN900 (Supplementary Table 4). However, only 2.5% (29/1,145) of mapped structures with less than 75 percent identity (PID) are sourced from *C. difficile*. Conversely, 85.5% (65/76) of mapped structures with greater than 75 PID are *C. difficile* specific (Supplementary Fig. 1). This steep drop off in the number of *C. difficile* mapped structures demonstrates the overall structural knowledge gap for *C. difficile*. Only 20 of the genes within iCN900 map to a structure that is greater than 75 PID and sourced from *C. difficile*. These represent the best characterized, metabolically related *C. difficile* specific structures^23–26^.

### Experimental validation of iCN900 demonstrates high model accuracy

**Fig. 2:**
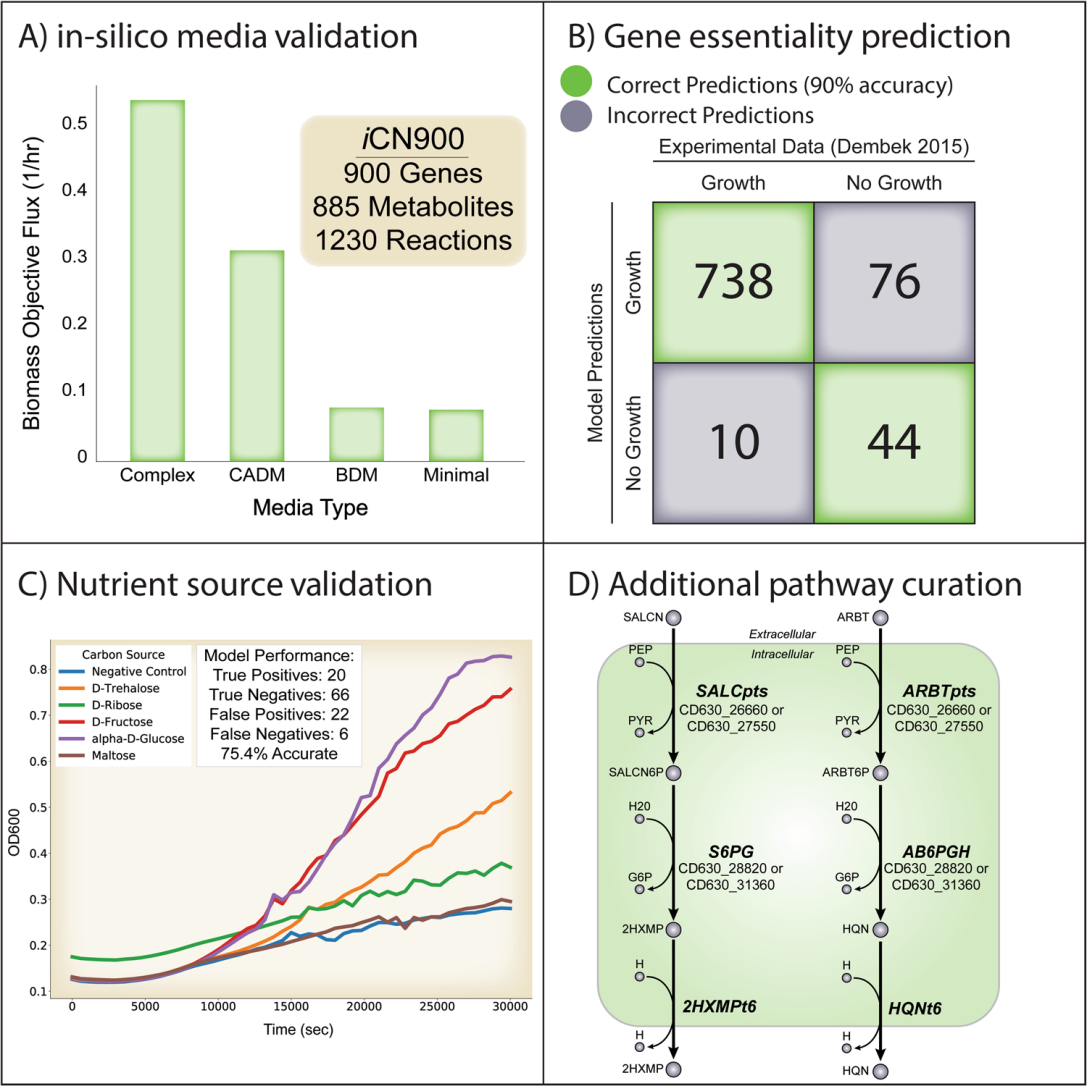
Properties and validation metrics of iCN900. (**A**) Model predictions for biomass flux on four different in silico media types: Complex media, CADM, BDM, and minimal media. Importantly, the biomass objective flux reflects the increasing amount of nutrients in each media condition. The overall gene, reaction, and metabolite content of iCN900 is summarized within the inset box. (**B**) Comparison of model predictions of essential genes on complex media compared to experimental gene-knockout results from Dembek et al. (**C**) *C. difficile* optical density at 620 nm was measured over time in Biolog Phenotype Microarray plates. Representative growth curves for the Savidge 630 strain on 5 indicated carbon sources (of the 190 tested) and the negative control are shown. Experimental growth of *C.* *difficile* was compared to iCN900 metabolic flux predictions, to determine the accuracy of predictions as summarized in the inset box. (**D**) Putative metabolic pathways for *C. difficile* utilization of salicin and arbutin were incorporated into iCN900 through targeted gap-filling enabled by comparison to experimental growth data.

We evaluated iCN900 by performing simulations on four in silico media types as delineated by Larocque et al.^10,11^ (1) minimal, (2) basal defined medium (BDM), (3) complete amino acid-defined medium (CADM) and (4) complex media. We confirmed biomass production by iCN900 under each in silico media type and further showed that flux through the objective function increased commensurate with the complexity of media type (Fig. 2A). We also confirmed that known essential amino acids required by *C. difficile* growth (cysteine, leucine, isoleucine, proline, tryptophan, and valine)^27,28^ are also required for biomass production.

To assess gene-essentiality prediction by iCN900, we performed in silico single gene deletions and compared these predictions to an available experimental dataset of essential genes for *C. difficile* in strain R20291^29^. *C. difficile* R20291 is evolutionarily distinct from strain 630 and the iCN900 model achieved an overall accuracy of 90% for these gene-essentiality predictions (Fig. 2B). The true negative gene predictions are predominantly associated with reactions encoding lipid metabolism indicating that in complex media this portion of the metabolic network is particularly sensitive to single gene knockouts both in silico and in vitro. Examining the ten false negative predictions revealed genes involved in pyrimidine metabolism indicating that perhaps R20291 has alternative encoding mechanisms for reactions in this pathway. Future improvements to CD630 reconstructions would benefit from experimentally validated gene-essentiality datasets specific to this strain.

Finally, we validated the ability of the iCN900 model to predict growth capabilities on 190 diverse carbon sources by comparing model predictions to the phenotypic microarray growth data generated for the three independent laboratory strains of *C. difficile* 630 (Fig. 2C, Supplementary Table 1). In silico growth predictions of the iCN900 model were generated using previously defined minimal media conditions (“Methods”) and alternating the carbon source to coordinate with that being tested in the experimental microarray^10,27^. Of the 190 carbon sources tested, 114 were represented in the model and overall model predictions agreed with experimental growth capabilities for 75.4% of cases (Supplementary Text 2).

### Targeted gap-filling of incorrect model predictions uncovers new catabolic pathways in *C. difficile* metabolism

Comparison of phenotypic screens to model predictions can be used to iteratively improve GEM reconstructions by informing the inclusion of metabolic pathways missing in the network content. Using the phenotypic microarray growth data generated from each *C. difficile* strain, we evaluated the phenotypic divergence of closely related strains against our curated iCN900 GEM. Our data confirmed previously published studies^30–32^ and verified growth of two of the three *C. difficile* 630 strains on salicin, arbutin, and N-acetyl-galactosamine (GalNAc) (Fig. 1A**)**. However, initially the iCN900 model predicted the inability of CD630 to grow on these compounds (Supplementary Text 3). Both salicin and arbutin are β-glucosides and are produced in various plant species thus it is plausible that these compounds could be available within the human gut dependent on diet^33,34^. We identified homologous genes in the pathways for catabolism of these two compounds in *Bacillus subtilis*, a close relative of *C. difficile*. Our identified candidate pathways have a similar pathway architecture: a transporter (encoded for by *ptsG-A* and *ptsI*), a glucohydrolase (encoded for by *celF* and *bglA7*), and efflux of 2-hydroxymethyl-phenol or hydroquinone, respectively, both products of the respective glucohydrolase. Homologs in the *C. difficile* 630 genome were identified and incorporated into iCN900 using gene product rules based on homology with *B. subtilis* (Fig. 2D) and the experimental evidence that these compounds support growth.

Like salicin and arbutin, our experimental growth assays verified N-acetyl-galactosamine was sufficient to support growth of two of the three *C. difficile* 630 strains tested, but this phenotype was absent from our initial rendition of the iCN900 model. N-acetyl-galactosamine is of particular interest because as a host-derived glycan it is proposed to be an important carbon and nitrogen source for *C. difficile* in the gastrointestinal tract^30^. We hypothesized that N-acetyl-galactosamine utilization would be facilitated by a phosphotransferase system (PTS) similar to those seen in other enteric bacteria and investigated other GEMs for N-acetyl-galactosamine catabolic pathways. We identified an isomerase encoded by *agaI* in *E. coli*^35^ that converts N-acetyl-galactosamine-6-phosphate to tagatose-6-phosphate. Our experimental dataset indicates all three *C. difficile* 630 strains grew significantly (*P* = 0.006, Paired *T*-Test) in the presence of tagatose (6.59 fold-change relative to negative control) but did not grow on galactose (0.89 fold-change), thus supporting the possibility of this interconversion. In agreement with the experimental results, iCN900 predicts *C. difficile* growth on tagatose (true positive), and no growth on galactose (true negative). This inference along with the strength of the experimental evidence led to the inclusion of the PTS and isomerase within iCN900. Further experimental work to identify any additional genes that encode this machinery would increase understanding of N-acetyl-galactosamine utilization by *C. difficile*, which may have important implications in the context of infection.

Surprisingly, iCN900 predicted an inability to be grown in ethanolamine, which is in contrast to our experimental evidence and the literature that many gut bacteria, including *Clostridia*, are capable of ethanolamine catabolism as a sole carbon or nitrogen source^36^. Furthermore, phosphatidylethanolamine is a prevalent membrane phospholipid, which is catabolized into glycerol and ethanolamine, suggesting that ethanolamine is an abundant nutrient in the gastrointestinal tract. iCN900 contains the genes of the *eutG* operon and the corresponding enzymes for usage of ethanolamine^37^. Previous studies have shown that *C. difficile* 630 strains can utilize ethanolamine in vitro, however, the media conditions in these studies included glucose along with ethanolamine^37^. We postulated that if phosphatidylethanolamine is a primary source of ethanolamine within the gut, then glycerol would be concurrently available to *C. difficile*. Interestingly, glycerol scored as a false positive in our initial prediction. Further analysis revealed that when both glycerol and ethanolamine were components of the in silico minimal media the biomass objective flux increased to 0.034 from 0.014 on glycerol alone or 0 on ethanolamine alone. This apparent synergistic usage predicted by iCN900 of these two metabolites is interesting given their likely co-availability in the host. Glycerol as a sole carbon source has a limited uptake flux value of 4.56 and valine and leucine were identified as non-carbon limiting nutrients. When both ethanolamine and glycerol are available both have an uptake flux of ten, indicating an energetically favorable complement of catabolic pathways. Ethanolamine utilization produces acetyl-CoA, which is a key metabolite in many downstream metabolic pathways. We hypothesize that the ability to use ethanolamine as a source to produce the necessary acyl-carrier proteins frees glycerol to be used for other growth requirements. While no modifications were made to the network to change the determination of glycerol as a false positive prediction and ethanolamine as a false negative prediction, it is worth noting this potential feature of *C. difficile* physiology and a future validation of this prediction would be valuable.

### iCN900 links observed mutations to unique phenotypes

With an updated reconstruction completed, we used this resource to evaluate the mutations and deletions observed between the three reference strains (Tables 1 and 2). Of the deleted genes, two are implicated in the metabolism of fructose and mannose that are of particular interest. First, Savidge 630 and Britton 630Δ*erm* each contain a deletion of CD630_02880, which is part of the GPRs for both fructose and mannose PTS reactions. Second, a unique deletion in Britton 630Δ*erm* of CD630_31350, a gene involved in the fructose bisphosphate aldolase reactions. Growth results reveal the maximum fold change in optical density during fructose utilization is 24.3% lower (*P* = 0.24, Paired *T*-Test) in Britton 630Δ*erm* versus Savidge 630, and 34.8% lower (*P* = 0.008) versus Britton 630. During mannose utilization, growth reduction is 35.1% (*P* = 0.1) and 40% (*P* = 0.03), respectively. While there is no significant decrease in growth on both sugars between Britton 630Δ*erm* and the Savidge strain, the decreases between Britton 630Δ*erm* and Britton 630 are both statistically significant. Given the co-occurrence of deletions in the transport systems for these sugars and fructose bisphosphate aldolase reactions, we hypothesize that the deletions together result in the observed growth reduction for the Britton 630Δ*erm* strain with perhaps the more consequential deletion being CD630_31350.

**Fig. 3:**
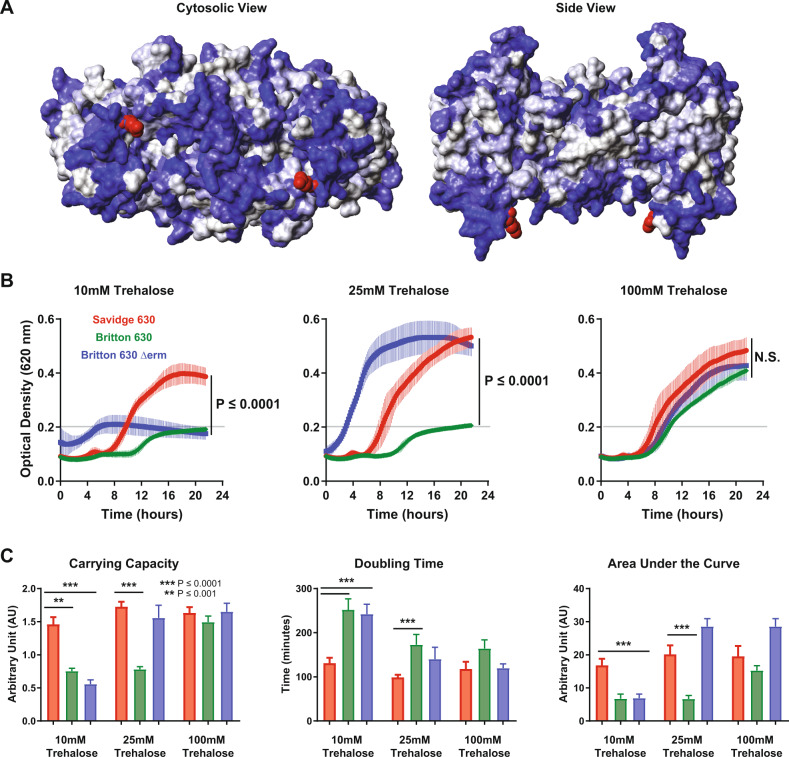
Characterization of phenotypic growth differences of lab-adapted isolates on trehalose. (**A**) Predicted protein structure of *C. difficile* PTS_(CD630_30890) based upon the crystal structure of the MalT transporter. The EIIC domain is shown as a dimer, with the E285D substitution of the 630-Savage isolate highlighted in red on the cytoplasmic interface. The model shading indicates amino acid hydrophobicity (gray residues are hydrophobic and blue residues are hydrophilic according to the Kyte-Dolittle scale). (**B**) Growth curves of *C. difficile* isolates, Savidge 630 (red), Britton 630 (green) and Britton 630Δerm (blue) in defined minimal medium supplemented with trehalose. The gray line indicates the maximal optical density of the negative control wells. Optical density at 620 nm measured at 10 min intervals, The plotted bar is the mean of three biological replicates assayed in duplicate wells and the error bars represent the standard deviation of the mean. (**C**) Growth curves from the conditions in (**B**) were analyzed by Gaussian process curve fitting to calculate the total carrying capacity, doubling time, and total area under the curve (error bars represent the standard deviation of the mean **P ≤ 0.001, ***P ≤ 0.0001).

Mutations within coding sequences and particularly those in genes annotated with metabolic functions were prioritized. The Savidge 630 strain possesses a substitution in the aspartate kinase gene (G68C (GGT → TGT)). However, there were no physiological changes in the growth experiments on aspartic acid, which is likely explained by the presence of aspartic acid in the basal medium. Savidge 630 also contained a unique nonsynonymous substitution (E258D (GAA → GAC)) in CD630_30890, which is part of the gene product rule for the trehalose phosphotransferase reaction. Analysis of the growth screen data indicated that the maximal optical density of the Savidge 630 strain during trehalose utilization was over 30% greater (*P* = 0.04) than either the Britton 630 or the Britton 630Δ*erm* strains. Mapping of this substitution to the predicted protein structure reveals that it occurs within a hydrophilic region of the protein (Fig. 3A), suggesting that the substitution may confer an advantage to the import or phosphorylation of trehalose entering the cell. To test this hypothesis, growth curves in minimal medium supplemented with 10, 25, and 100 mM trehalose were compared (Fig. 3B), revealing that at the lower concentration of trehalose the Savidge 630 strain grew significantly better than the other two strains (*P* ≤ 0.0001). However, in higher concentrations of trehalose, the growth of the Britton 630Δ*erm* isolate (25 mM) and the Britton 630 isolate (100 mM) matched that of Savidge 630. The significant increase in growth at 10 mM supplementation of trehalose indicates that the substitution may increase affinity of the PTS for trehalose transport, improve efficiency of transport, or increase expression and/or stability of the transporter however the role of the this E258D substitution in trehalose uptake still needs to be explored. Analysis of the growth curves by Gaussian Process modeling^38^ allowed us to quantify growth rate, area under the curve, and carrying capacity of the isolates in each condition (Fig. 3C).

Both the Savidge 630 strain and the Britton 630Δ*erm* strain had unique mutations within the CD630_26670 gene, which codes for part of the PTS reaction for α-glucose. In the Savidge 630 strain the single-nucleotide polymorphism results in a substitution of isoleucine for valine (V228I (GTT → ATT)), however, the mutation in the Britton 630Δ*erm* strain switches the stop codon to glutamic acid (*524E (TAA → GAA)). The loss of this stop codon in the Britton 630Δ*erm* strain results in extension of the CD630_26670 coding region directly into the downstream gene with the next stop codon at position 691. The lack of a stop codon would likely produce an aberrant transcript subject to degradation by cellular regulatory mechanisms^39^. Analysis of the growth data supports the hypothesis these substitutions impair the import of α-glucose as growth via maximum optical density of the Savidge 630 strain is reduced by 21.7% and the Britton 630Δ*erm* strain is reduced by 30.4% compared to Britton 630 strain (*P* = 0.05), which is devoid of any mutations in these genes. Overall, the mutational analysis provides insight into unintentional evolution occurring in laboratory strains and highlights the need for resequencing strains used commonly across many labs to more accurately reflect the heterogeneity among reference sequences. This is particularly important for the accuracy of corresponding GEMs and downstream constraints-based analyses.

### iCN900 applied to analyze sequence variation within the *C. difficile* core-genome

**Fig. 4:**
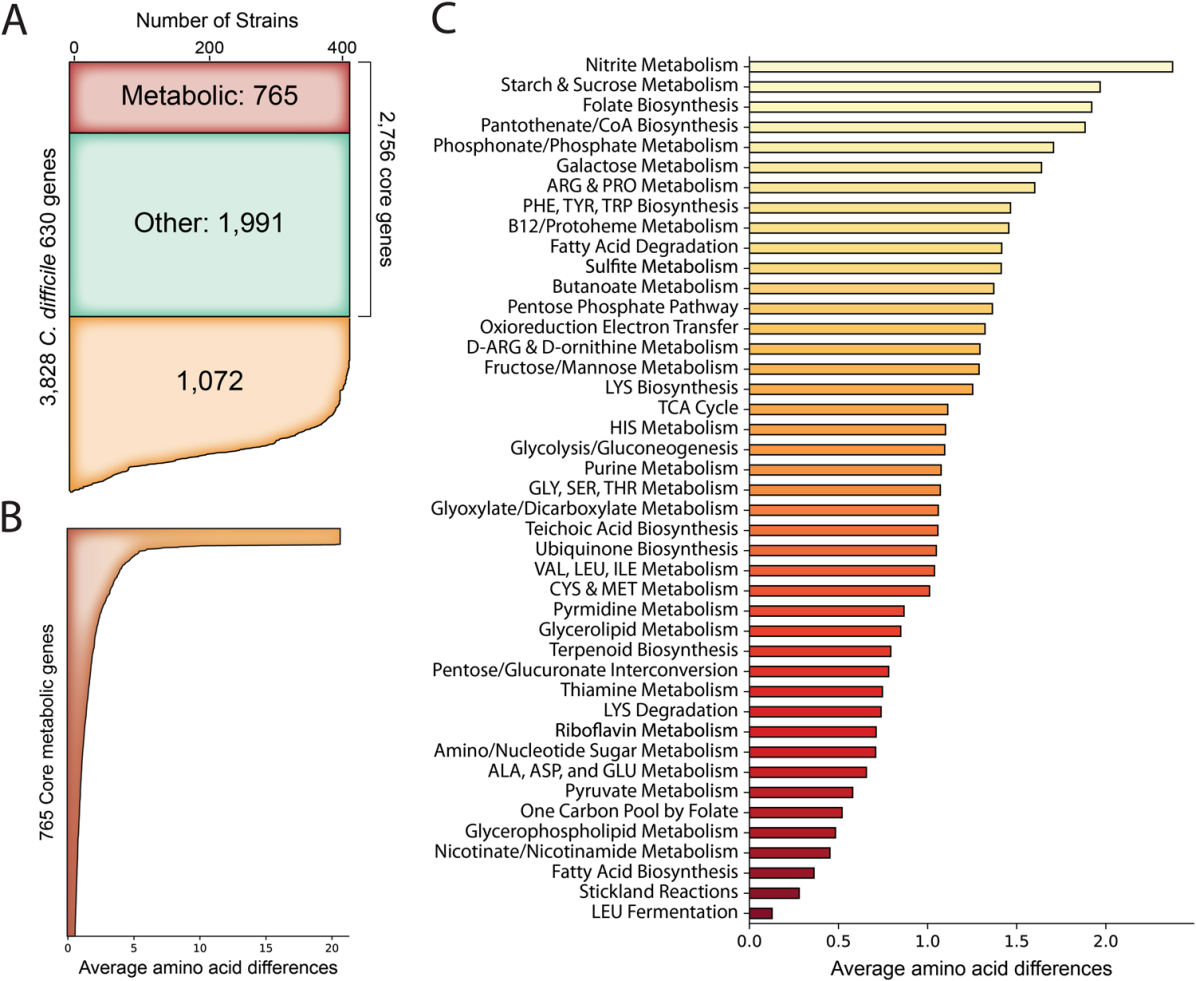
Core-genome of *C. difficile* reveals metabolic subsystems with greater sequence variation. (**A**) By comparing the genomes of 415 publicly available *C. difficile* genomes the core-genome was calculated and includes 765 metabolic genes. (**B**) Analyzing the sequence variation among the 765 core metabolic genes demonstrates that the average difference in amino acid sequence range from 0 to just over 20 for these shared genes. (**C**) The genome-scale reconstruction enables stratification of the genes by metabolic subsystem and comparison of average amino acid differences of each gene within a subsystem. This reveals that nitrite and starch/sucrose metabolism have the highest degree of sequence variation, whereas Stickland reactions and leucine fermentation are the most conserved.

We used the iCN900 model to link mutations amongst the three strains to the differences observed within the phenotypic growth profiles. iCN900 is specific to *C. difficile* 630, one of the most well characterized strains and often used as a reference strain in studies. However, we have shown that there is genetic divergence within even 630 stock cultures from different laboratories. As demonstrated above, single-nucleotide variations can manifest themselves as deviations in metabolic profiles pointing to the importance of even small amounts of genetic divergence between *C. difficile* isolates. Therefore it is worth considering the sequence variation amongst shared genes within several strains of the species. To this end we used bi-directional BLAST to identify the genes within *C. difficile* 630 present at greater than 80 PID in 415 high-quality, publicly available genomes (Fig. 4A). From these genes, those that were present in more than 99% (411/415) of the strains were determined to comprise the core-genome of *C. difficile*. A total of 2756 of 3828 *C. difficile* 630 genes comprise the core-genome (Supplementary Data File 5). iCN900 was then utilized to investigate the metabolic core-genome which consisted of 765 core metabolic genes. A GEM based on the function of these 765 genes was created to investigate core *C. difficile* metabolic capabilities; iCN765 (Supplementary Data File 6). This representation of the core metabolic functions of the *C. difficile* species represents a potentially valuable starting point for reconstruction of other strains. The core model was used to investigate metabolic phenotypes common to all strains of *C. difficile*. Simulations with in silico minimal media predict that the core metabolic network cannot produce biomass. However, media supplementations were identified that enable synthesis of certain biomass constituents. Protein synthesis required supplementation with histidine, lysine, arginine, and threonine. Supplementation with uridine or uracil enabled DNA and RNA synthesis and nicotinate supplementation enabled associated cofactor production. Following these media supplementations the core network still lacks the ability to produce the lipid and peptidoglycan biomass components. Performing gene-essentiality analysis on the full *C. difficile* 630 model using this supplemented in silico media condition predicts that there are four non-core genes which are essential for the production of lipids and peptidoglycan. Upon further examination, three of these genes are present within 96% (398/415) of the strains, thereby designated as non-core and perhaps the strains without these genes have either acquired alternative encoding mechanisms or vary in lipid/peptidoglycan composition. It is worth noting that the strains without these three genes represent the strains of type MLST11 and MLST254 within the group of 415. The final non-core gene essential to production of peptidoglycan is only present within 13% (54/415) of strains and is involved in the production of teichoic acid for cell wall synthesis.

**Fig. 5:**
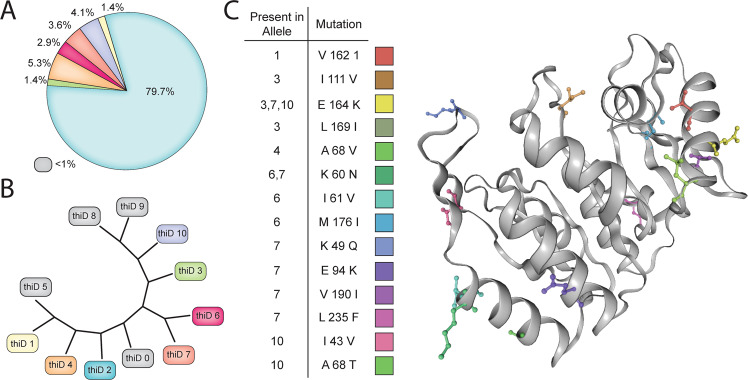
Allele diversity for *thiD* as an example of sequence diversity. (**A**) The 415 sequences for the *thiD* gene have 11 variant sequences (alleles) variably present within the population. Notably the reference sequence allele is present within 79.7% of the population, whereas the next most frequent allele is present in 5.3% of the population. (**B**) The degree of similarity between each sequence is readily accessible. For example the *thiD* 6 and *thiD* 7 sequences are similar to one another sharing a K60N mutation. (**C**) Through the use of the GEM-PRO each mutation by variant can be visualized within the 3D space of crystal structures where applicable.

Beyond investigating conserved metabolic functions, examining the conserved sequences amongst the 415 strains provides other novel insights (Fig. 4B). In the core metabolic gene products, we evaluated the average amino acid difference and found them to range from zero (completely conserved amino acid sequence across all strains) to just over 20 average amino acid differences between strains. For example, we identified strain FDAARGOS_268 (PATRIC ID:1496.2022) with the same trehalose phosphotransferase (CD630_30890) E258D mutation described in Savidge 630 above as well as strain QCD-32g58 (PATRIC ID: 367459.5) with an E258K substitution in the same protein. Strain QCD-32g58 was isolated in 2017 from a patient in Quebec, Canada with severe CDI and is noted to be a representative of a predominant Quebec strain. Furthermore, the greatest average amino acid differences (>20 average amino acid differences) occurred in two gene products, CD630_01370 and CD630_35270, that are implicated in transport reactions for cellobiose and iron, respectively. Each individual gene can also be interrogated for the frequency of each allele sequence within the group of 415 strains (Fig. 5A) and these sequences can be compared for their similarity to one another (Fig. 5B). For the genes that are part of the GEM-PRO the mutations per allele can be mapped to the representative structure providing a three dimensional view of the effect of the change (Fig. 5C). We performed this analysis for the *thiD* gene encoding phosphomethylpyrimidine kinase and gained insight into the areas of the protein structure where the sequence variants manifested.

The GEM-PRO also allows for a systems-level analysis of the variation within these core-gene products by stratifying the average amino acid differences per reaction to metabolic subsystems (Fig. 4C). This network context illuminates the metabolic subsystems that may be under evolutionary selective pressures due to higher degrees of sequence variation. The reactions for nitrite metabolism, starch and sucrose metabolism, and folate biosynthesis have the greatest variation indicating these are potential evolutionary hotspots. Conversely, leucine fermentation and Stickland reactions are the most conserved in terms of sequence suggesting that these enzymes and related functions are defining traits within the species.

To increase the analysis of metabolic network areas that may be under selective pressure within *C. difficile*, we considered the classification of enzyme specificity. Generally, it is understood that specificity is an evolutionarily beneficial trait toward increased catalytic efficiency. We used iCN900 to classify the genes and reactions within as either generalist or specialist. As previously reported^40^ we define a specialist gene as one that participates in only one reaction and generalists as those involved in multiple reactions. We applied this criteria to all metabolic enzymes within and showed that there are 410 specialist genes encoding proteins catalyzing 287 specialist reactions and 231 generalist genes encoding proteins catalyzing 484 reactions. This distribution is similar to that previously found for *E. coli*^40^. Of the specialist genes, 76 encode subunits of a complex and 148 are isozymes. Similar to our analysis of the sequence variation of the core-genome, we used the reconstruction to evaluate the distribution of specialist and generalist reactions per metabolic subsystem. Analyzing each subsystem we found that certain subsystems were enriched in specialist enzymes and others in generalist enzymes. Starch and sucrose metabolism, folate biosynthesis, vitamin B12 and protoheme metabolism, and histidine metabolism are all enriched in specialist reactions (hypergeometric *P* < 0.05). Valine, leucine and isoleucine metabolism, glycerolipid metabolism, one carbon pool by folate, and fatty acid biosynthesis are all enriched in generalist reactions (hypergeometric *P* < 0.05). Consistent with the calculation of sequence variation amongst subsystems the specialist enriched subsystems had an average of 1.61 amino acid differences and the generalist enriched subsystems had an average of .69 average amino acid differences (Supplementary Fig. 2). These network based analyses enabled by the reconstruction provide insights into the pressure surrounding the core metabolism of *C. difficile* as a species and point to vulnerable processes worth investigating as potential drug targets.

## Discussion

Genome scale metabolic network reconstructions provide a valuable format to unify disparate knowledge about an organism, and contribute a tool that may be used to investigate an organism’s properties. We developed the most comprehensive knowledge base for *C. difficile* strain 630 to date and utilized the model to (1) investigate catabolic capabilities in conjunction with experimental data; (2) serve as a framework for investigation into genetic drift amongst different laboratory *C. difficile* 630 strains and a derivative strain; (3) analyze the sequence variation amongst the genes within the core-genome of *C. difficile*. The GEM performs with as much as 90% accuracy in predicting gene essentiality and 75% accuracy in predicting catabolic capabilities. The metabolic network represented within iCN900 is devoid of EGCs and the standardization of reaction and metabolite identifiers opens up the possibility of inclusion in studies of multiple organisms that share this namespace. Phenotypic profiling and model driven discovery identified new pathways potentially relevant to *C. difficile* survival due to their presence in the diet (arbutin and salicin) or as components of the human gut (N-acetyl-galactosamine). By coupling the generation of the new reconstruction, iCN900, with extensive phenotypic profiling and further genome analytics we have increased the body of knowledge about this pathogen.

The process of crafting iCN900 evoked questions of genetic drift amongst isolates of the same strain of bacteria. The variability in both genotype and phenotype of isolates that are either deemed strain 630 or are closely related points to the need to resequence strains used in experiments and to recognize that reference sequences represent only a single time-point in the lifetime of a strain. This point was borne out in our comparison of the trehalose transporter between laboratory strains. Hypervirulent strains of *C. difficile* are known to metabolize trehalose, a process recently attributed to hypervirulent strain evolution coinciding with the widespread adoption of trehalose in our diet^41^. Microevolution of strains within laboratories could impart divergent conclusions between laboratories undergoing similar experimental processes to evaluate pathogen evolution and virulence, which may serve to hinder translational science and limit new treatment options. This phenomenon has been observed in other model organisms including *E. coli*^5^ and yeast^42^. In *E. coli, glpR* mutations have been observed leading to constitutive expression of genes involved in glycerol catabolism likely due to repeated passage on glycerol containing media. Similar unexpected *glpR* alleles have been found in several other *E. coli* strains^43^. Thus a similar process of unintentional domestication of laboratory *C. difficile* strains based on adaptation to laboratory media may be underway. Given the importance of metabolism in infection kinetics and virulence, diligence in tracking genetic drift within strains will collectively improve scientific rigor and reproducibility with the potential to strengthen bodies of scientific evidence between laboratories.

Motivated by the demonstrated divergence in metabolic profile from small amounts of genetic diversity, the core-genome of *C. difficile* was constructed based on 415 publicly available genome sequences and sequence variation was analyzed. The reconstruction was used to identify metabolic traits common to the species and amino acid differences and enzyme specificity were used to evaluate which pieces of the metabolic network may be under selection pressures and those that are more conserved. Interestingly, and in agreement with the growing literature^41,44,45^ concerning sugar metabolism of pathogenic *C. difficile* strains this analysis revealed that even conserved starch and sucrose metabolism genes are some of the most varied in terms of sequence. This demonstrates that *C. difficile* strains are actively evolving more efficient machinery to best adapt to their nutrient niche (be it in a lab or in the colon) and that unique catabolic capabilities could arise in response to availability of certain nutrients.

The generation of this high-quality reconstruction enables future studies extrapolating this model across multiple strains to investigate species diversity. While we focused on core metabolic capabilities in this study, the exploration of accessory metabolic gene sets are underway and could give insight into the metabolic capacity specific to hypervirulent strain families of *C. difficile*. The ability to identify evolutionary hotspots and specialized enzymatic reactions within hypervirulent strains may help direct drug development targeting previously unappreciated metabolic processes critical to pathogen survival. Furthermore, the ability to simulate coordinated changes in dietary supplements and predicted evolutionary hotspots could give insight into pathogen emergence.

## Methods

### Reconstruction

We began the reconstruction of iCN900 by using previous efforts iMLTC806cdf^10^ and icdf834^11^ for *C. difficile* strain 630. This starting point was refined and translated to a reconstruction within the standardized BiGG namespace. This reconstruction was then extensively manually curated. In addition, evaluation metrics as delineated in a protocol for generating reconstructions were executed^9^. Model content was iteratively improved by comparison to existing and generated experimental data. iCN900 reflects the final version of this iterative workflow.

### Constraint-based modeling

Constraints-based analyses were conducted using the COBRApy toolbox. For the in silico growth simulation of sole carbon source utilization the minimal media^27^ was used and glucose was removed in an iterative fashion and other carbon source exchange reactions were opened to evaluate if growth was possible. Growth versus no growth was determined through FBA in each condition, optimizing for the biomass function. Within these simulations we consider biomass objective flux of greater than zero designated carbon sources that supported growth.

### Protein structure integration

The GEM-PRO^21,22^ pipeline was used to annotate iCN900 with available protein structure information. The list of genes within iCN900 was mapped to sequences within Uniprot and consequently the Uniprot ID enables automatic mapping to the PDB. The representative sequences are then BLASTed to the PDB and the best ranking structure available was identified for each model gene was identified and the quality of those rankings are presented.

### Core-genome

A total of 1246 whole-genome sequences of *C. difficile* were downloaded from the PATRIC database^46^ on August 25, 2019. To filter for high-quality genomes a cutoff of assemblies composed of 100 or fewer contigs was applied. Furthermore, an MLST analysis of the genomes was performed using MLST^47,48^. All genomes that could not be assigned to an MLST type or species were also filtered out. This led to a final set of 415 genome sequences for downstream analysis.

### Designation of specialist and generalist enzymes

We classified 697 metabolic enzymes within iCN900 as either specialists or generalists. The selection criteria was a simplified approach as presented within^40^ as the supplementary information to refine the approach is not as well defined for *C. difficile* as for *E. coli*. The 697 genes to be classified were selected from the reconstruction on the basis that they are not involved in any transport reactions. Following the definition of the group each was classified according to the following rule; specialist if the gene is present within the GPR of only one reaction and generalist for those involved in more than one reaction. In turn it was possible to classify the encoded reactions in a corresponding manner as either specialist or generalist. The reaction classifications were then analyzed according to their metabolic subsystems and each subsystem was tested for enrichment of either class through the hypergeometric test.

### Whole-genome sequencing

Cryofrozen isolates of each *C. difficile* strain were incubated on Brain Heart Infusion (BHI) agar under anaerobic conditions for 24–48 h. Genomic DNA was extracted using the MasterPure Complete DNA & RNA Purification kit (Lucigen, MC85200) and libraries of fragmented genomic DNA were prepared using NEXTflex Rapid DNA-Seq Kit (Bioo Scientific, NOVA-5149-02). Paired-end reads (2 × 150 bp reads) were generated on the MiSeq platform (Illumina, San Diego, CA, USA) using the Illumina MiSeq Reagent Kit v2 (MS-102-2002) and PhiX Control Kit v3 (FC-110-3001). Breseq v0.31^13^ was run with default parameters on each set of paired-end reads with the *C. difficle* 630 genome (AM180355.1) as a reference. We note that the individual CD630 strains utilized within this study have each been subcultured within their respective labs over time. The Britton 630 strain was received from a colleague at Tufts University on July 23, 2008 and the Savidge 630 strain was received from a colleague at the University of Houston in August 2014. Further we note that the Britton 630 strain is not the parent strain to Britton 630Δ*erm*.

### Phenotypic profiling by biolog

Strains were cultured in BHI medium (Difco) supplemented with 0.5% (w/v) yeast extract (Fischer Scientific) overnight (~16 h) in an anaerobic chamber (5% hydrogen, 90% nitrogen, 5% carbon dioxide). One milliliter of overnight culture was diluted into 10 ml of defined minimal media with previously described composition (Theriot et al, 2017) and 100 µl was added to each well of Biolog Phenotypic Microarray plates (PM1 and PM2). Growth assays were performed under anaerobic conditions with optical density at 620 nm read every 10 min over a period of 16 h, in triplicate for each *C. difficile* 630 strain. Statistical analysis was performed by two-way ANOVA, (with Tukey’s correction for multiple comparisons where appropriate) in GraphPad Prism Software (v. 7.04).

### Reporting summary

Further information on research design is available in the Nature Research Reporting Summary linked to this article.

## Supplementary information

Supplementary Table 5

Supplementary Table 4

Reporting Summary

Supplementary Information

Supplementary Table 1

Supplementary Table 2

Supplementary Table 3

Supplementary Dataset 1

Supplementary Dataset 2

Supplementary Dataset 3

Supplementary Dataset 4

Supplementary Dataset 5

Supplementary Dataset 6

## Data Availability

All data generated in this study are included with this published article and associated supplementary data files. iCN900 is additionally available on the BiGG Models Database. Three laboratory stock strain sequences used in this study are available on NCBI with the BioProject accession number PRJNA649005.
