# Supplementary material for: Systems biology analysis of the Clostridioides difficile core-genome contextualizes microenvironmental evolutionary pressures leading to genotypic and phenotypic divergence
Source: NPJ Syst Biol Appl. 2020 Oct 20;6:31. doi: 10.1038/s41540-020-00151-9 (PMC7576604; doi:10.1038/s41540-020-00151-9)
Supplement: Supplementary file 4 — Supplementary Information [file 41540_2020_151_MOESM4_ESM.pdf]

Supplementary Figures:

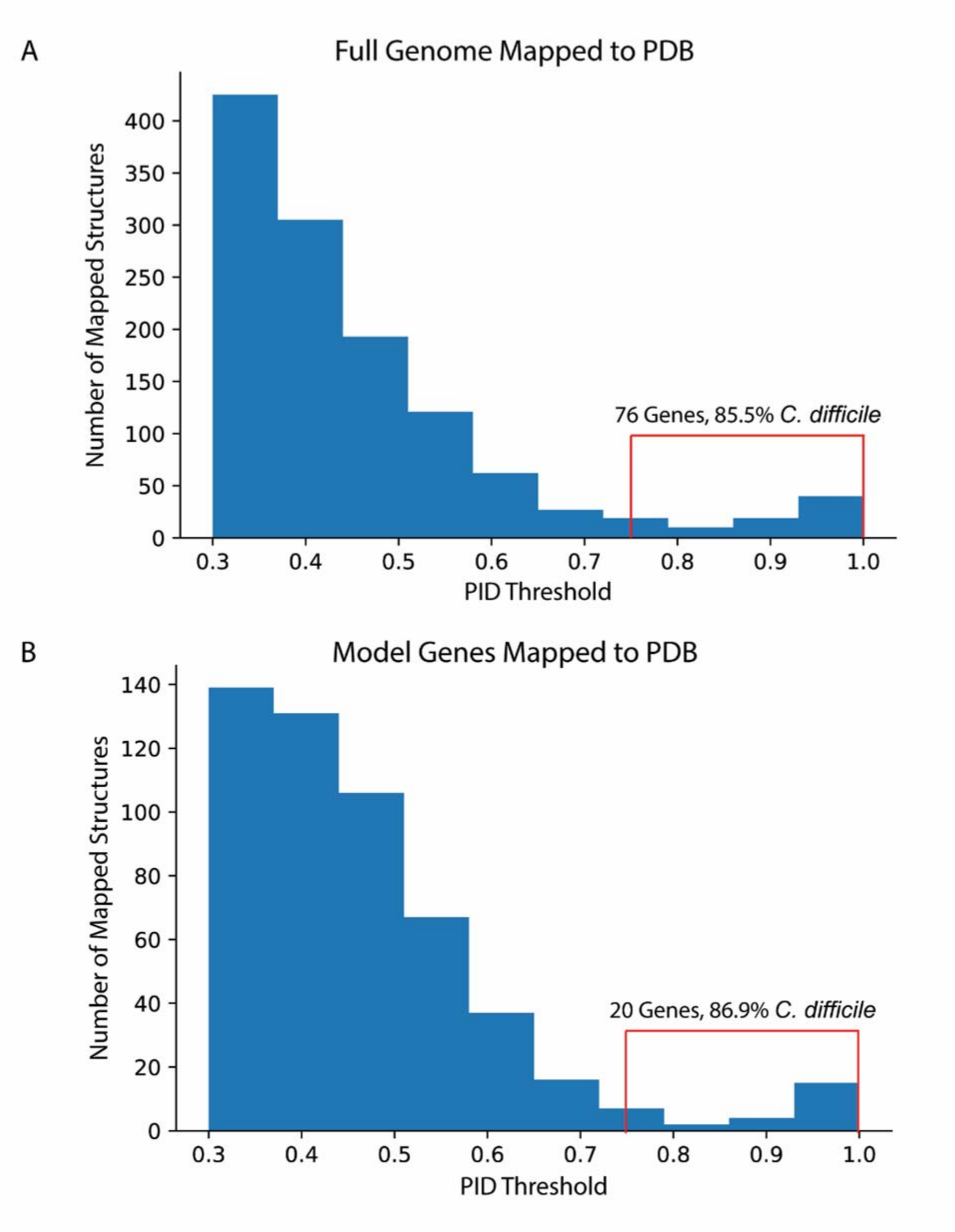

Supplementary Figure 1: Histogram detailing the amount of genes mapped to the PDB within the full reference genome and within iCN900 model genes

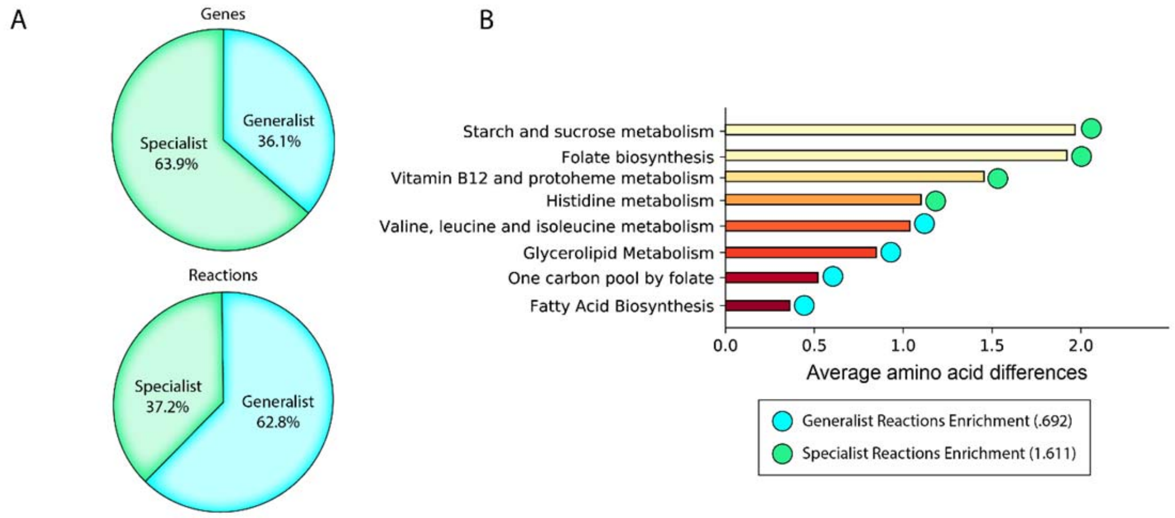

Supplementary Figure 2: A) Pie chart detailing the percentage of genes and reactions designated as either specialists or generalists. B) Each subsystem was checked for significant enrichment via hypergeometric test and the subsystems with enrichments are shown along with the corresponding average amino acid sequence variation.

Supplementary Data:

Supplementary Dataset 1: Breseq Reports for each strain
Supplementary Dataset 2: Reconstruction in spreadsheet format iCN900.ods
Supplementary Dataset 3: Reconstruction in json format iCN900.json
Supplementary Dataset 4: Reconstruction in sbml format iCN900.xml
Supplementary Dataset 5: Full PID for each 630 gene across the 415 strains used to construct the core-genome.
Supplementary Data File 6: Reconstruction of core metabolism of C. difficile iCN765.json

Supplementary Text:

Supplementary Text 1. Model Reconstruction Process

We began the network reconstruction by evaluating both the existing GEMs for *C. difficile* 630: iMLTC806cdf and icdf834. The first reconstruction produced by Laroque et. al in 2014 included 806 genes, 1,013 reactions, and 703 metabolites and represented the first effort at a manually curated network reconstruction for *C. difficile* [10]. In addition to the first curated network this work included validation of the model on four types of *in silico* media, identification of essential amino acids, and evaluation in comparison to an automatically generated network. In 2017 Kashaf et al. produced icdf834 a second GEM that improved upon iMLTC806cdf [11]. icdf834 includes 834 genes, 1227 reactions, and 807 metabolites and the major expansion of content is reflected in the inclusion of fatty acid, glycerolipid, and glycerophospholipid pathways. Overall, iMLTC806cdf and icdf834 provided a valuable starting point and iCN900 represents the next step in this lineage providing increases in both network quality and content.

To expedite the process of improving and adding to the network we first translated the previous efforts to standardized BiGG format for reaction and metabolite identifiers [19,20]. By putting the model into a standardized notation the tractability of the network has been greatly improved and

now the iCN900 is a part of a large repository of GEMs in BiGG notation that includes a diverse phylogeny of organisms [49–54]. The slight drop in reaction number from icdf834 to iCN900 is a result of changing the duplicate secretion and exchange reaction set up in icdf834 to a more conventional set of single exchange reactions. We standardized the previous networks to BiGG [19,20] reaction and metabolite identifiers to increase the usability of the reconstruction [55]. We also subjected the previous curated network to rigorous validation and removed the presence of many erroneous energy generating cycles. Building upon the now robust version of the metabolic network derived from the foundation of iMLTC806cdf and icdf834 we added a significant amount of new content to the reconstruction. As a means of further quality assurance of iCN900 we ran the reconstruction through the MEMOTE test suite for consistency [56]. iCN900 scores 100% on the metrics of charge balance, metabolite connectivity, and checks for unbounded flux in default medium. iCN900 scores 87.1% for mass balance and 17% for stoichiometric consistency. We hypothesized that the lack of mass information for some components of the lipid metabolism results in this issue. These reactions have not been fully characterized and the ambiguous stoichiometry likely results in this one particularly low score. To test this we created a version of the model excluding these such reactions and saw the expected increase in the score for stoichiometric consistency. Thus this is less an issue of the reconstruction and represents a current knowledge gap on the composition of these metabolites and reaction stoichiometries.

iCN900 contains an additional 66 genes, 46 reactions, and 70 metabolites versus the content present in icdf834 (**Supplementary Table 2**). These additions were made through a variety of techniques including use of the annotation tool DETECT v2 [14], BLAST with the most closely related reconstructions [15] , and curation of pathways based on false negative model predictions against experimental data. DETECT v2 is an enzyme annotation tool that assigns potential enzyme commission number to protein sequence. We ran DETECT v2 on the reference genome for *C. difficile* 630 and extensively looked through the results cross-referencing with the genome annotation [57] to find a number of new genes with predicted metabolic function that could be added to the model. This proved to be a valuable method for identifying candidate new reactions and corresponding gene product rules (GPRs), but necessitated rigorous examination of the automatic results to ensure accuracy. The second means for adding content to the reconstruction was by utilizing BLAST to identify homologous genes with *Bacillus subtilis* and *Clostridium ljungdahlii*. These organisms were chosen on the basis that they are the most closely related organisms for which there exists a high-quality GEM [53,54]. Utilizing the homologous genes and GPRs from these reconstructions we were able to fill gaps in the previous *C. difficile* network. Encouragingly, many of the genes that were eventually added were identified independently in both the DETECT v2 and BLAST homology based workflows. Lastly, there were a number of compounds from experiments with Biolog Phenotypic Microarrays that the model originally incorrectly predicted unable to sustain growth. These false negative predictions provide opportunity for further network curation since the experimental data suggests the organism has the necessary machinery to grow on these compounds. Thus iCN900 includes reaction content that reconciles three key false negative model predictions for salicin, arbutin, and N-acetyl-galactosamine into agreement with the experimental data (Figure 1A and 2D). The reactions included for each are SALCpts and S6PG, ARBTpts and AB6PGH, and ACGALpts and ACGAL6PI, which are all gene annotated with the exception of ACGAL6PI.

Supplementary Text 2. Model Validation

Essential gene predictions were performed as had been done previously with iMLTC806cdf and icdf834. Critically, in the evaluation of icdf834, Kashaf et al. utilize the experimental dataset of essential genes for *C. difficile* R20291 that had been generated in the intervening time between

iMLTC806cdf and icdf834 [29]. The switch from comparison to *Bacillus subtilis* essential gene data was conducted originally for iMLTC806cdf was a significant improvement.

iCN900 had 90% accuracy prediction of essential genes compared to the Dembek dataset. While Kashaf et al. report an accuracy for icdf834 of 92.3% they calculate their accuracy only on the predicted model essential genes and not the full confusion matrix of homologous genes. We reran gene essentiality predictions with icdf834 and found the overall accuracy in comparison to the R20291 experimental data to also be 90%, but for a smaller number of homologous genes since iCN900 reflects an increase in gene content. Overall the predictions made with iCN900 are based on 868 homologous genes to R20291 and evaluation of the full confusion matrix results in a Matthews correlation coefficient of .504.

Of the 190 compounds screened in the assay 114 of them directly map to metabolites within the BiGG database. Each of the profiled strains was compared to the *in silico* predicted growth capabilities and resulted in the following accuracies on tractable metabolites: 74.56%, 72.8%, and 67.5%. If the assumption is made that the model would predict no growth on the compounds that do not map to BiGG and are therefore not included within the model, then the accuracies increase to 81%, 80%, and 78%. Overall, iCN900 demonstrates a high prediction accuracy to the phenotypic data.

Supplementary Text 3. Further False Negative and False Positive Predictions

Leucine and methionine were also classified originally as false negatives, but we hypothesize that this is an artifact of recapitulating the proprietary Biolog media as defined *in silico* media. As both these metabolites are already within the minimal media, we looked at the relative biomass yield if the amount of either of these compounds was increased. From this analysis we found that increasing the leucine available resulted in increased biomass yield whereas increased methionine did not. As such leucine may be considered a true positive by the model and methionine remains a false negative. Additionally D-arabitol is likely a false negative that is more accurately considered a true negative prediction as the fold change in OD from the Biolog experiment is right at the threshold in our analyses for what we consider growth. This is supported by the fact that the growth values for L-arabitol definitively show no growth. There are two remaining false negative model predictions that could not be rectified to true positives that merit further analysis: succinate and ethanolamine.

Previous studies suggest that *C. difficile* 630 can utilize succinate as a carbon source through the usage of succinate to butyrate pathway [58]. While iCN900 includes the aforementioned pathway and corresponding supplemental pathways such as sorbitol fermentation pathway, thought to provide complimentary electron flow, the model predicts no growth when succinate is the sole carbon source in minimal media. It is worth noting that the addition of a succinate dehydrogenase using ubiquinone as a cofactor would enable growth on succinate, however there is no compelling genetic basis for this reaction and therefore it was not added.

Supplementary Tables:

Supplementary Table 1: Biolog data for each strain

Xpt Compound	Savidge CD630	Savidge CD630	Savidge CD630	Britton CD630	Britton CD630	Britton CD630	Britton CD630 Δerm	Britton CD630 Δerm	Britton CD630 Δerm
L-Arabanose	1.2	1.2	1.1	0.8	1.1	1.1	0.9	0.9	0.9
N-Acetyl-D-Glucosamine	4.2	2.4	4.1	4.3	4.1	4.0	3.2	3.6	3.2
D-Saccharic Acid	1.4	1.0	1.0	1.3	1.0	1.1	1.0	0.3	0.8
Succinic Acid	2.0	1.8	1.5	1.6	1.5	1.7	1.1	0.4	1.2
D-Galactose	0.9	1.1	0.9	0.9	0.9	1.1	0.8	0.4	1.0
L-Aspartic Acid	1.1	1.0	1.2	0.9	1.2	1.0	0.9	0.3	1.0
L-Proline	2.5	1.5	1.5	0.9	1.1	1.0	0.9	0.4	1.0
D-Alanine	0.8	1.0	1.1	0.8	1.1	1.0	0.9	0.3	1.0
D-Trehalose	5.1	2.8	4.2	2.0	4.2	1.8	2.7	2.2	2.9
D-Manose	3.9	2.6	4.6	4.3	4.6	3.1	2.7	2.4	2.0
Dulcitol	0.5	0.8	0.9	0.9	0.9	1.1	0.9	0.3	0.9
D-Serine	1.4	1.1	0.7	0.6	0.7	1.1	0.9	0.4	1.1
D-Sorbitol	5.4	2.3	2.1	3.3	2.1	2.9	1.6	1.6	2.4
Glycerol	1.2	1.0	0.7	0.8	0.7	1.0	1.0	0.3	0.9
L-Fucose	1.3	1.0	0.6	0.7	0.6	1.1	0.8	0.3	0.8
D-Glucuronic Acid	1.3	1.0	0.6	0.8	0.6	1.0	0.9	0.3	0.9
D-Gluconic Acid	1.2	0.9	0.7	0.8	0.7	1.1	0.9	0.4	1.0
D,L-alpha-GlycerolPhosphate	1.3	0.9	0.7	0.8	0.7	1.0	0.9	0.5	1.0
D-Xylose	1.3	1.4	1.3	0.8	1.1	1.3	1.0	0.2	1.2
L-Lactic Acid	1.2	1.0	0.7	0.8	0.7	0.9	0.8	1.0	1.0
Formic Acid	1.2	1.0	0.9	0.6	0.9	1.1	0.9	0.4	1.0
D-Mannitol	4.7	2.4	3.9	4.0	3.9	3.8	2.6	2.3	2.7
L-Glutamic Acid	1.2	0.7	0.7	0.6	0.7	1.0	0.9	0.3	1.0
D-Glucose-6-Phospate	1.1	0.8	0.7	0.6	0.7	1.0	0.9	0.5	1.0
D-Galactonic Acid-gamma-Lactone	1.2	0.9	0.6	0.5	0.6	1.0	0.8	0.7	0.9
D,L-Malic Acid	0.9	0.9	0.6	0.7	0.6	1.0	0.8	0.6	1.0
D-Ribose	1.7	1.6	1.7	2.0	1.7	1.8	1.0	0.3	1.1
Tween 20	1.0	0.9	0.5	0.9	0.5	1.0	0.7	1.0	0.8
L-Rhamnose	1.1	1.0	0.6	0.6	0.6	1.2	0.7	0.4	0.9
D-Fructose	4.2	2.6	4.4	4.3	4.4	4.3	3.0	2.2	3.3
Acetic Acit	0.7	0.9	0.7	0.6	0.7	1.0	0.8	0.8	0.9
alpha-D-Glucose	4.2	2.5	4.9	4.6	4.9	4.4	3.6	2.4	3.7
Maltose	1.3	1.0	0.6	0.6	0.6	1.0	0.8	0.5	0.9
D-Melibiose	1.1	1.0	0.7	0.7	0.7	1.0	0.7	0.3	0.9
Thymidine	1.7	0.6	0.8	0.8	0.8	1.3	0.9	0.4	1.1
L-Asparagine	0.9	0.8	0.6	0.6	0.6	1.0	0.8	0.5	1.0

D-Aspartic Acid	0.9	0.8	0.5	0.7	0.5	1.0	0.8	0.3	0.8
D-Glucosaminic Acid	1.1	0.8	0.7	0.7	0.7	1.1	0.8	0.2	1.1
1,2-Propanediol	1.2	0.8	0.7	0.7	0.7	1.2	0.8	0.4	1.0
Tween 40	0.9	0.9	0.8	0.8	0.8	1.0	0.7	0.3	0.8
alpha-Keto-Glutaric Acid	1.1	0.9	0.4	0.4	0.4	1.2	0.8	0.3	0.9
alpha-Keto-Butyric Acid	1.7	1.8	1.8	1.2	0.8	1.0	1.1	0.3	1.3
alpha-Methyl-D-Galactoside	0.9	0.9	0.6	0.7	0.6	1.0	0.8	0.5	0.9
alpha-D-Lactose	1.2	0.9	0.6	0.7	0.6	1.0	0.7	0.2	0.9
Lactulose	0.9	0.9	0.6	0.6	0.6	1.1	0.8	0.7	0.9
Sucrose	0.9	1.0	0.6	0.7	0.6	1.1	0.8	0.2	0.8
Uridine	1.6	1.0	0.7	0.7	0.7	1.2	0.8	0.3	1.0
L-Glutamine	0.8	0.8	0.6	0.7	0.6	1.1	0.8	0.5	0.9
m-Tartaric Acid	1.0	0.8	0.7	0.7	0.7	1.0	0.8	0.2	0.9
D-Glucose-1-Phosphate	1.1	0.8	0.6	0.7	0.6	1.0	0.8	0.3	0.9
D-Fructose-6-Phosphate	1.2	0.9	0.6	0.7	0.6	1.0	0.8	0.3	0.9
Tween 80	0.7	0.6	0.6	0.8	0.6	1.0	0.7	0.3	0.8
alpha-Hydroxy Glutaric Acid-gamma-Lactone	1.0	0.9	0.6	0.7	0.6	1.0	0.8	0.3	1.0
alpha-Hydroxy Butyric Acid	2.1	1.2	0.7	0.9	0.7	1.3	0.9	0.3	1.0
beta-Methyl-D Glucoside	1.1	0.5	0.6	0.6	0.6	0.8	0.5	0.4	0.5
Adonitol	0.5	0.8	0.5	0.5	0.5	0.9	0.8	0.3	0.9
Maltotriose	1.1	1.0	0.6	0.7	0.6	1.0	0.7	0.2	0.8
2-Deoxy Adenosine	0.3	0.7	0.1	0.3	0.1	0.8	0.8	0.2	0.9
Adenosine	1.3	1.1	0.7	0.9	0.7	1.3	1.0	0.4	1.1
Glycyl-L-Aspartic Acid	1.4	0.8	0.7	0.6	0.7	0.8	0.7	1.1	0.9
Citric Acid	1.0	0.7	0.6	0.6	0.6	0.9	0.7	0.8	0.9
m-Inositol	0.9	0.8	0.6	0.7	0.6	1.0	0.7	0.4	0.8
D-Threonine	0.7	0.8	0.6	0.5	0.6	1.1	0.7	0.4	0.9
Fumaric Acid	0.5	0.8	0.5	0.6	0.5	0.8	0.8	0.3	0.9
Bromo Succinic Acid	0.7	0.9	0.6	0.7	0.6	0.7	0.8	0.4	0.9
Propionic Acid	0.6	0.9	0.6	0.7	0.6	0.9	0.8	0.6	1.0
Mucic Acid	0.7	0.9	0.6	0.7	0.6	0.9	0.8	0.5	0.9
Glycolic Acid	0.8	0.9	0.6	0.7	0.6	0.9	0.8	0.4	0.9
Glyoxylic Acid	0.3	0.6	0.2	0.1	0.2	0.6	0.4	0.4	0.5
D-Cellobiose	1.5	1.7	1.7	0.6	0.7	1.1	0.7	0.3	0.8
Inosine	0.8	1.0	0.6	0.7	0.6	1.1	0.9	0.2	1.0
Glycyl-LGlutamic Acid	0.8	0.8	0.7	0.7	0.7	0.8	0.7	0.4	0.9

Tricarballylic Acid	1.0	0.8	0.7	0.7	0.7	1.0	0.6	1.0	0.9
L-Serine	2.1	1.3	1.7	1.0	1.2	1.2	1.1	0.5	1.3
L-Threonine	2.0	1.3	1.3	1.4	1.3	1.3	1.6	1.7	1.5
L-Alanine	0.5	0.8	0.6	0.6	0.6	0.8	0.7	0.3	0.8
L-Alanyl-Glycine	1.3	0.8	0.6	0.6	0.6	0.9	0.7	0.3	0.9
Acetoacetic Acid	0.7	0.8	0.6	0.6	0.6	0.9	0.7	0.4	0.9
N-Acetyl-beta-D Mannosamine	2.3	1.6	1.7	0.7	0.7	1.4	0.8	0.4	1.0
Mono Methyl Succinate	1.4	1.2	0.7	0.7	0.7	1.3	0.9	0.3	1.0
Methyl Pyruvate	1.1	1.3	0.2	0.8	0.2	1.2	1.3	0.5	1.1
D-Malic Acid	0.6	0.4	0.7	0.7	0.7	0.9	0.8	0.5	0.9
L-Malic Acid	0.8	0.9	0.7	0.7	0.7	0.9	0.9	1.1	0.9
Glycyl-L-Proline	1.6	1.1	0.7	0.6	0.7	0.5	0.8	0.5	1.0
p-Hydroxy Phenyl Acetic Acid	0.7	0.7	0.7	0.7	0.7	0.9	0.9	0.2	1.0
m-Hydroxy Phenyl Acetic Acid	1.3	0.8	0.7	0.6	0.7	1.1	0.9	0.4	1.1
Tyramine	0.5	0.8	0.4	0.3	0.4	0.7	0.9	0.5	0.9
D-Psicose	1.0	0.9	0.8	0.7	0.8	1.1	0.9	0.2	1.1
L-Lyxose	0.8	0.9	0.7	0.4	0.7	0.7	0.6	0.3	0.8
Glucuronamide	0.7	0.8	0.8	0.6	0.8	0.7	0.8	0.7	1.0
Pyruvic Acid	1.1	1.1	0.5	0.4	0.5	1.1	0.9	0.2	1.1
L-Galactonic Acid-gamma-Lactone	0.7	0.9	0.7	0.7	0.7	0.6	0.8	0.5	1.0
D-Galacturonic Acid	0.7	0.9	0.6	0.6	0.6	0.9	0.7	0.4	0.8
Phenylethylamine	0.4	0.4	0.1	0.1	0.1	0.3	0.7	0.4	0.9
2-Aminoethanol	1.1	1.1	1.0	1.8	2.0	3.0	1.7	1.8	1.6
Chondroitin Sulfate C	0.6	0.5	0.9	0.5	0.9	0.6	0.1	0.0	0.5
alpha-Cyclodextrin	0.9	0.8	1.0	0.8	1.0	1.0	0.2	0.0	0.5
beta-Cyclodextrin	1.0	0.7	1.3	0.7	1.3	0.9	0.3	0.1	0.6
gamma-Cyclodextrin	0.9	0.8	1.2	0.8	1.2	0.9	0.2	0.5	0.5
Dextrin	0.8	0.7	1.1	0.7	1.1	0.9	0.1	0.1	0.5
Gelatin	0.9	0.7	1.0	0.7	1.0	0.9	0.3	0.1	0.6
Glycogen	0.9	0.7	1.0	0.7	1.0	0.9	0.1	0.3	0.4
Inulin	0.9	0.7	1.1	0.7	1.1	0.9	0.6	0.7	0.9
Laminarin	1.5	1.5	1.7	1.8	1.7	1.9	0.5	1.1	0.8
Mannan	0.9	0.7	1.1	0.7	1.1	0.9	0.1	0.1	0.4
Pectin	0.9	1.0	1.2	1.0	1.2	1.1	1.5	1.7	1.8
N-Acetyl-DGalactosamine	1.5	1.6	1.8	1.6	1.8	1.8	0.3	0.1	0.6
N-AcetylNeuraminic Acid	2.3	2.2	2.4	2.2	2.4	2.4	0.3	0.1	0.6
beta-D-Allose	0.6	0.8	0.7	0.8	0.7	1.0	0.2	0.0	0.5

Amygdalin	0.5	0.8	0.4	0.8	0.4	0.9	0.3	0.1	0.6
D-Arabinose	1.1	0.9	1.2	0.9	1.2	1.1	4.4	2.4	3.7
D-Arabitol	1.2	1.0	1.4	1.0	1.3	1.2	0.2	0.0	0.5
L-Arabitol	0.9	1.0	0.5	1.0	0.5	1.2	0.2	0.0	0.5
Arbutin	3.1	3.6	4.4	3.6	4.4	3.7	0.3	0.1	0.6
2-Deoxy-D Ribose	0.1	0.2	0.1	0.2	0.1	0.4	0.2	0.1	0.5
i-Erythritol	0.6	0.7	0.7	0.7	0.7	0.9	0.3	0.0	0.6
D-Fucose	1.0	0.8	1.1	0.8	1.1	1.0	0.2	0.0	0.5
3-0-beta-D-Galactopyranosyl-D Arabinose	0.6	0.7	1.1	0.7	1.1	0.9	0.2	0.0	0.5
Gentiobiose	0.8	0.8	1.1	0.8	1.1	1.0	0.2	0.0	0.5
L-Glucose	0.9	0.9	1.1	0.9	1.1	1.1	0.1	0.0	0.4
Lactitol	0.9	0.9	1.2	0.9	1.2	1.0	0.2	0.0	0.5
D-Melezitose	3.9	4.3	4.9	4.3	4.9	4.5	0.2	0.0	0.5
Maltitol	0.9	0.8	1.1	0.8	1.1	1.0	0.2	0.1	0.5
a-Methyl-D Glucoside	0.6	0.8	0.4	0.8	0.4	1.0	0.2	0.0	0.5
beta-Methyl-DGalactoside	0.8	0.8	0.8	0.8	0.8	0.9	0.2	0.0	0.5
3-Methyl Glucose	0.7	0.8	0.6	0.8	0.6	0.9	0.2	0.0	0.5
beta-Methyl-DGlucuronic Acid	0.9	0.9	1.1	0.9	1.1	1.0	0.2	0.0	0.5
alpha-Methyl-DMannoside	0.8	0.8	1.1	0.8	1.1	0.9	1.2	0.0	1.5
beta-Methyl-DXyloside	1.0	0.9	1.2	0.9	1.2	1.0	0.2	0.0	0.5
Palatinose	0.9	0.8	1.3	0.8	1.3	1.0	3.3	2.0	3.6
D-Raffinose	0.9	0.8	1.3	0.8	1.3	1.0	0.3	0.1	0.6
Salicin	2.8	2.8	3.6	2.8	3.6	3.0	0.3	0.0	0.6
Sedoheptulosan	0.7	0.8	0.8	0.8	0.8	1.0	0.2	0.1	0.5
L-Sorbose	0.6	0.8	0.5	0.8	0.5	1.0	0.2	0.0	0.5
Stachyose	0.9	0.8	1.3	0.8	1.3	0.9	0.2	0.0	0.5
D-Tagatose	3.3	3.6	4.3	3.6	4.3	3.7	16.2	4.1	16.5
Turanose	1.0	0.9	1.3	0.9	1.3	1.1	0.2	0.1	0.5
Xylitol	0.5	1.0	0.3	1.0	0.3	1.2	0.2	0.0	0.5
N-Acetyl-D Glucosaminitol	0.6	0.7	0.7	0.7	0.7	0.9	0.3	0.0	0.6
gamma-Amino Butyric Acid	0.7	0.8	0.6	0.8	0.6	1.0	0.2	0.0	0.5
delta-Amino Valeric Acid	0.8	0.8	0.8	0.8	0.8	1.0	0.2	0.0	0.5
Butyric Acid	0.9	0.8	1.1	0.8	1.1	1.0	0.2	0.1	0.5
Capric Acid	0.7	0.4	0.3	0.4	0.3	0.6	0.4	0.1	0.7
Caproic Acid	0.8	0.9	1.1	0.9	1.1	1.1	0.3	0.1	0.6
Citraconic Acid	0.9	1.0	1.2	1.0	1.2	1.2	0.2	0.1	0.5
Citramalic Acid	0.6	0.9	0.6	0.9	0.6	1.1	0.2	0.1	0.5
D-Glucosamine	2.8	3.1	3.7	3.1	3.7	3.3	3.3	2.4	3.6

2-Hydroxy Benzoic Acid	0.5	0.8	0.6	0.8	0.6	1.0	0.2	0.1	0.5
4-Hydroxy Benzoic Acid	0.7	0.9	0.9	0.9	0.9	1.1	0.1	0.1	0.4
beta-Hydroxy Butyric Acid	1.2	1.2	1.0	1.2	1.0	1.3	0.2	0.0	0.5
gamma-Hydroxy Butyric Acid	1.1	0.9	0.6	0.9	0.6	1.1	0.2	0.0	0.5
a-Keto-Valeric Acid	1.6	1.8	1.5	1.8	1.6	1.9	0.2	0.1	0.5
Itaconic Acid	0.6	0.7	0.9	0.7	0.9	0.8	0.2	0.0	0.5
5-Keto-D Gluconic Acid	1.0	0.9	1.0	0.9	1.0	1.1	0.5	1.1	0.8
D-Lactic Acid Methyl Ester	0.9	0.9	1.0	0.9	1.0	1.1	0.3	0.1	0.6
Malonic Acid	0.8	0.9	1.1	0.9	1.1	1.1	0.2	0.1	0.5
Melibionic Acid	0.7	0.7	0.9	0.7	0.9	0.9	0.5	0.1	0.8
Oxalic Acid	0.7	0.8	1.0	0.8	1.0	1.0	0.1	0.0	0.4
Oxalomalic Acid	0.8	0.9	0.8	0.9	0.8	1.0	0.2	0.1	0.5
Quinic Acid	0.8	0.9	0.7	0.9	0.7	1.0	0.2	0.1	0.5
D-Ribono-1,4-Lactone	0.8	0.9	0.7	0.9	0.7	1.1	0.1	0.0	0.4
Sebacic Acid	0.8	0.8	0.7	0.8	0.7	1.0	0.2	0.0	0.5
Sorbic Acid	0.2	0.4	0.3	0.4	0.3	0.6	0.3	0.1	0.6
Succinamic Acid	0.8	0.9	0.5	0.9	0.5	1.1	0.2	0.0	0.5
D-Tartaric Acid	0.8	0.8	0.8	0.8	0.8	1.0	0.1	0.0	0.4
L-Tartaric Acid	0.9	0.8	1.1	0.8	1.1	1.0	0.1	0.0	0.5
Acetamide	0.7	0.9	0.9	0.9	0.9	1.0	0.3	0.1	0.6
L-Alaninamide	0.9	0.9	0.9	0.9	0.9	1.0	0.3	0.1	0.6
N-Acetyl-LGlutamic Acid	0.8	0.8	0.6	0.8	0.6	1.0	0.2	0.1	0.5
L-Arginine	0.9	0.8	0.5	0.8	0.5	0.9	0.2	0.1	0.5
Glycine	0.6	0.7	0.7	0.7	0.7	0.9	0.2	0.0	0.5
L-Histidine	0.9	0.8	0.7	0.8	0.7	1.0	0.2	0.0	0.5
L-Homoserine	0.4	0.8	0.5	0.8	0.5	0.9	0.2	0.0	0.5
Hydroxy-LProline	1.8	1.8	2.5	1.8	2.5	2.0	0.1	0.0	0.4
L-Isoleucine	0.5	0.6	0.5	0.6	0.5	0.7	0.3	0.2	0.6
L-Leucine	1.5	3.5	2.4	3.5	2.4	3.6	0.6	0.1	0.9
L-Lysine	0.8	0.8	1.1	0.8	1.1	1.0	0.3	0.1	0.6
L-Methionine	1.3	1.2	1.1	1.2	1.1	1.3	0.2	0.1	0.5
L-Ornithine	0.9	0.8	0.8	0.8	0.8	1.0	1.8	1.6	2.1
L-Phenylalanine	0.5	0.7	0.9	0.7	0.9	0.9	0.2	0.1	0.5
L-Pyroglutamic Acid	0.5	0.8	0.6	0.8	0.6	1.0	0.3	0.1	0.6
L-Valine	0.6	0.7	0.5	0.7	0.5	0.9	0.2	0.1	0.5
D,L-Carnitine	1.1	0.8	0.8	0.8	0.8	0.9	0.2	0.1	0.5
Sec-Butylamine	0.7	0.7	1.1	0.7	1.1	0.9	0.2	0.1	0.5
D.L-Octopamine	0.8	0.8	0.5	0.8	0.5	1.0	0.2	0.1	0.5

Putrescine	0.7	0.7	0.7	0.7	0.7	0.8	0.2	0.0	0.5
Dihydroxy Acetone	0.4	0.7	0.7	0.7	0.7	0.9	2.7	1.7	3.0
2,3-Butanediol	0.8	0.8	0.8	0.8	0.8	1.0	0.2	0.1	0.5
2,3-Butanedione	0.2	0.6	0.3	0.6	0.3	0.8	0.6	0.2	0.9
3-Hydroxy 2-Butanone	0.7	1.1	1.1	1.1	1.1	1.2	0.3	0.4	0.6

Supplementary Table 2: 66 new model genes and corresponding encoded reactions.

**Reaction**	**Gene Product Rule (GPR)**
Updated Existing GPR	
ATPS4r	(CD630_34700 and CD630_34740 and CD630_34720 and CD630_34710 and CD630_34690 and CD630_34680 and CD630_29570 and CD630_29600 and CD630_34760) or (CD630_34700 and CD630_34740 and CD630_34720 and CD630_34710 and CD630_34690 and CD630_34680 and CD630_34730 and CD630_29600 and CD630_34760 and CD630_02510)
AEPPYRTA	(CD630_28490 and CD630_07400)
TREpts	CD630_13360 and CD630_30890
BUTKr	(CD630_01130) or (CD630_23790) or (CD630_011301) and CD630_24260
UAGPT3	CD630_27250
FRUpts	(CD630_31340) or (CD630_02060 and CD630_02070 and CD630_02080) or (CD630_32780 and CD630_32790 and CD630_32770 and CD630_32760) or (CD630_10740 and CD630_10760 and CD630_10770 and CD630_10780) or (CD630_04910 and CD630_04920 and CD630_04930 and CD630_04940) or (CD630_02860 and CD630_02870 and CD630_02880 and CD630_02890) or (CD630_02840 and CD630_02850 and CD630_22690) or (CD630_36290 and CD630_36300)
MCOATA	(CD630_11810 or CD630_10620)
SBTpts	(CD630_07640 and CD630_07650 and CD630_07670) or (CD630_24140 and CD630_07650 and CD630_07670 and CD630_07660)
POR_syn	(CD630_01180 and CD630_21990 and CD630_21980 and CD630_26820)
PMPK	(CD630_13530) or (CD630_15590) or CD630_15990
AHSERL	(CD630_18250) and CD630_15940
SHK3Dr	(CD630_27080) or (CD630_270801) or CD630_18370
New Reactions	
SERTRS2	CD630_00140
PROTRS	CD630_00490 and CD630_00500
GLNTRS	CD630_00510 and CD630_20590
CYSTRS	CD630_00520
THRTRS	CD630_05740
ALATRS	CD630_12820
TYRTRS	CD630_15210
ASNTRS	CD630_22450

GLYTRS	CD630_24320 and CD630_24330
LEUTRS	CD630_25210
FMETTRS	CD630_25840
TRPTRS	CD630_26100
ASPTRS	CD630_27390
VALTRS	CD630_32560
METTRS	CD630_35400
SERTRS	CD630_00140
PHETRS	CD630_06990
GLCS1	CD630_08840
GLCP	CD630_08850
P5CR	CD630_14950 or CD630_32810
SERAT	CD630_15950
SPODM	CD630_16310
ADD	CD630_18200
Cuabc	CD630_21150
GLBRAN2	CD630_25260
PAPPT3	CD630_26540
UDPGD	CD630_27710
ACM6PH	CD630_30460
CYTOM	CD630_31470 or CD630_09270
FE3abc	CD630_35270 and CD630_35300
BGLA1	CD630_31360
METSR_S1	CD630_21660
MALTATr	CD630_08720
ACGApts	CD630_31370
CELBpts	CD630_27550 and CD630_36480 and CD630_36470
CODH_ACS	CD630_07300
CYSS	CD630_15940
MOBDabc	CD630_08700 and CD630_08680
MTHFR5	CD630_07210
26DPAi	CD630_29670
ACTNabc	CD630_19470
ASPR	CD630_22540
ANHMK	CD630_30510
MOGDS	CD630_17090

Supplementary Table 3: Energy Generating Cycles and Changed reactions to remedy them

Group of Metabolites with EGCs	Reaction Reversibilities Edited
ATP, CTP, GTP, UTP, ITP	LACLt2, POR_syn, PPDK, GLYCK, DATCY, DGTUP, DGTCY, URIK2, CYTDK2, DATUP, OAADC, PHHL2, GD3P2
NADH, NADPH,	FNRR, FNRR2, NITR, MTHFD, TRSARr, TRSARr2, FMNRx2,

FMNH2	
Q8H2, MQL8	HAAOR, GAPD, VOR2bE, SULR_syn

Supplementary Table 4: Model genes that map to a corresponding structure in the PDB sorted by PID

Gene Name	PDB ID	PID	PDB Chain ID
CD630_34260	4e16	1	A
CD630_18370	5dzs	1	B
CD630_34630	4lus	1	D
CD630_27330	4dgt	1	B
CD630_10550	5ol2	1	E
CD630_03970	3o3n	1	A
CD630_21890	4gib	1	B
CD630_10590	4dd5	1	A
CD630_10560	5ol2	0.985119	A
CD630_03980	3o3o	0.968	B
CD630_03960	4ehu	0.962406	A
CD630_22170	3js3	0.952941	B
CD630_10540	5ol2	0.952381	C
CD630_15990	4jjp	0.943182	B
CD630_22300	3tdr	0.937984	C
CD630_08720	3srt	0.924324	B
CD630_16310	4jzg	0.888889	A
CD630_19790	4nmy	0.878338	B
CD630_08910	3lhl	0.866438	A
CD630_30140	2m1z	0.849057	A
CD630_16530	4ote	0.841509	B
CD630_04460	3koz	0.780654	A
CD630_19130	2qez	0.770925	F
CD630_23410	1u8v	0.748996	C
CD630_31740	5jy6	0.743284	B
CD630_31700	4a3r	0.732558	D
CD630_07180	5a5g	0.729391	A
CD630_29550	3vr4	0.724289	E
CD630_34790	1i5e	0.717703	B
CD630_23350	1ak5	0.715431	A
CD630_14780	2k5l	0.712329	A
CD630_27100	2g0w	0.705674	B
CD630_34700	5hkk	0.702	A
CD630_00910	5g3y	0.699074	A
CD630_36410	3nzr	0.686992	A
CD630_25950	4p81	0.677966	D

CD630_16260	4fu0	0.669399	B
CD630_32850	3ifs	0.668151	B
CD630_17670	4dbv	0.662722	O
CD630_07590	1h18	0.660834	B
CD630_28820	1s6y	0.66055	A
CD630_10580	6acq	0.654804	F
CD630_22410	4utt	0.654709	B
CD630_27260	3pgy	0.652174	B
CD630_30640	1a0c	0.647191	B
CD630_04450	3kox	0.637097	G
CD630_14490	4uqf	0.636842	G
CD630_22780	1jdi	0.631579	B
CD630_31360	3pn8	0.628931	B
CD630_16970	1rvv	0.627451	1
CD630_08640	6dux	0.626697	A
CD630_30600	6dvv	0.625282	A
CD630_07520	2olj	0.625	B
CD630_01300	5h9u	0.624685	B
CD630_27450	4lza	0.623529	B
CD630_29560	1vdz	0.621622	A
CD630_13820	1bu6	0.621032	Y
CD630_07680	5o3z	0.618677	B
CD630_15650	4tsk	0.617021	A
CD630_31720	4y8f	0.615385	A
CD630_01010	4mki	0.614583	B
CD630_33170	1fdi	0.613445	A
CD630_11750	2iir	0.613065	C
CD630_26820	6cip	0.609839	B
CD630_11840	4ls8	0.606796	B
CD630_26910	2geb	0.605714	A
CD630_23390	3gk7	0.604598	A
CD630_24100	1kc7	0.603429	A
CD630_24330	5f5w	0.60274	E
CD630_10620	5h9g	0.6	A
CD630_35140	1dkr	0.598101	A
CD630_00480	5iwx	0.596273	B
CD630_01230	3sg1	0.595745	A
CD630_19170	3k9d	0.595092	A
CD630_00140	2dq3	0.593381	A
CD630_01790	2yfq	0.591449	A
CD630_27140	3enk	0.590504	B
CD630_16910	6gnb	0.583051	A
CD630_30960	2xhy	0.582278	B

CD630_32230	3ird	0.580205	A
CD630_31730	1php	0.58	A
CD630_21560	4wcx	0.576419	A
CD630_32250	3a5f	0.576271	B
CD630_15530	4evz	0.575397	B
CD630_31710	1o98	0.57451	A
CD630_10330	3beo	0.572193	A
CD630_32680	4gd5	0.571429	A
CD630_08410	2b1g	0.570332	D
CD630_01530	2yaj	0.569845	C
CD630_16630	2i9u	0.567376	B
CD630_34800	3ph3	0.566667	B
CD630_02190	3nua	0.564655	B
CD630_21380	1z9d	0.564103	C
CD630_35550	2h3g	0.5625	X
CD630_22540	3ojc	0.561404	D
CD630_36220	2akl	0.561404	A
CD630_23290	3r8r	0.560185	B
CD630_18990	2hvw	0.558621	A
CD630_26100	5v0i	0.558559	A
CD630_10570	5z7r	0.558491	B
CD630_21720	4ymw	0.558233	A
CD630_32370	6hje	0.558209	B
CD630_32810	3gt0	0.558052	A
CD630_05800	1qi6	0.557613	B
CD630_11530	6f2c	0.554745	D
CD630_28840	4mge	0.553398	A
CD630_15920	6hrb	0.550365	B
CD630_27550	2hro	0.549123	A
CD630_36550	4m0g	0.547786	A
CD630_19380	2vpq	0.543046	A
CD630_25750	1rpx	0.542601	A
CD630_20300	4jqo	0.541176	C
CD630_17920	5z75	0.540881	D
CD630_01130	1x9j	0.54039	B
CD630_00210	3ho8	0.538933	A
CD630_03990	5ol2	0.538462	F
CD630_08000	5z7r	0.538462	B
CD630_12250	3h5q	0.538117	A
CD630_17850	1j20	0.536341	A
CD630_29940	4dr0	0.535604	B
CD630_14950	3gt0	0.535581	A
CD630_24910	1qwr	0.535032	B

CD630_31240	4f66	0.534591	B
CD630_15340	2nlz	0.534451	D
CD630_26780	5dbn	0.533937	B
CD630_30460	4m0d	0.532895	C
CD630_25210	2byt	0.531017	D
CD630_11800	2z6j	0.530744	B
CD630_03890	4ipl	0.530738	B
CD630_15370	4ylf	0.528017	B
CD630_10050	5cee	0.527919	A
CD630_35350	4xb6	0.525952	D
CD630_12230	3m8z	0.525641	C
CD630_29670	3mcu	0.52551	D
CD630_05580	2pby	0.524272	B
CD630_28800	3k1s	0.52381	D
CD630_09910	3vba	0.521472	E
CD630_15120	3mxt	0.521277	A
CD630_35520	3a74	0.520629	D
CD630_03240	5x41	0.52	M
CD630_31840	5ygr	0.519802	C
CD630_01980	5tw7	0.518591	D
CD630_30910	5brq	0.517117	D
CD630_35980	1j6v	0.516556	A
CD630_13340	5nxa	0.515593	G
CD630_16580	1wyv	0.515464	H
CD630_23700	5hup	0.514815	F
CD630_07290	1htp	0.512	A
CD630_04510	3r6a	0.508333	A
CD630_19370	2f9i	0.507042	B
CD630_28130	1vpd	0.506757	A
CD630_14500	3h21	0.505618	A
CD630_14080	3r5x	0.505017	A
CD630_29950	6cgl	0.505007	A
CD630_27390	1c0a	0.504202	A
CD630_23430	2g39	0.501946	B
CD630_14530	3pg9	0.501916	B
CD630_15500	2f1d	0.5	P
CD630_20880	4zoh	0.5	C
CD630_02210	2z01	0.5	A
CD630_21280	3noy	0.498575	A
CD630_04870	2w1v	0.498182	B
CD630_00150	1wwr	0.496689	C
CD630_24380	1jtk	0.496241	B
CD630_20850	5ygr	0.49505	A

CD630_23200	3hee	0.493243	A
CD630_20320	2v5h	0.493007	E
CD630_01280	5buy	0.492958	B
CD630_02240	2yw2	0.492788	A
CD630_06990	4p73	0.492625	D
CD630_32240	2qz9	0.492492	B
CD630_14910	4k3f	0.490494	A
CD630_15480	2vd2	0.490385	A
CD630_13860	2phc	0.489177	B
CD630_12240	3la8	0.488889	A
CD630_31280	4gib	0.488789	A
CD630_00500	1h4s	0.486486	B
CD630_17040	1xm3	0.484375	D
CD630_25000	1tj7	0.484018	A
CD630_00520	1li5	0.483871	B
CD630_19470	2pcj	0.482456	A
CD630_02180	2ywx	0.481013	A
CD630_04030	1rv8	0.480519	C
CD630_02080	2m1z	0.480392	A
CD630_30060	3ox4	0.480315	D
CD630_35150	4aaw	0.479303	A
CD630_35770	5m3z	0.479167	A
CD630_25580	1vlh	0.478788	F
CD630_00370	1ik6	0.478659	A
CD630_21880	1h54	0.478318	A
CD630_13390	2gb3	0.477387	D
CD630_21680	1oa0	0.477358	B
CD630_07410	1bot	0.476378	O
CD630_01200	4amv	0.47541	C
CD630_12050	5e8l	0.474576	B
CD630_21300	5dul	0.473958	A
CD630_27080	3tnl	0.473868	B
CD630_19070	3zdr	0.473404	A
CD630_18260	2ghr	0.472973	A
CD630_11810	3ptw	0.471519	A
CD630_16980	4i14	0.471033	A
CD630_09890	1sr9	0.47069	A
CD630_32270	3r8y	0.470588	F
CD630_07280	1ru3	0.470339	A
CD630_23310	3h2z	0.469974	A
CD630_05740	1qf6	0.469484	A
CD630_20830	1yny	0.469298	A
CD630_32310	4qyi	0.469274	D

CD630_23790	1x9j	0.469101	H
CD630_01190	6gyz	0.46875	A
CD630_23300	2fxv	0.468421	B
CD630_25880	3tau	0.468293	B
CD630_19360	2f9i	0.468153	A
CD630_22760	6cse	0.468132	C
CD630_02200	1ao0	0.468132	B
CD630_02510	5b0o	0.46697	B
CD630_31780	4kir	0.466523	A
CD630_13380	6dux	0.466368	B
CD630_28280	3ele	0.466165	B
CD630_35890	1jdb	0.464183	L
CD630_01860	5ue9	0.463333	A
CD630_25260	4bzy	0.463268	A
CD630_26950	4lns	0.462462	A
CD630_02220	3auf	0.461929	A
CD630_18320	4grs	0.459941	B
CD630_26770	1k6d	0.458716	B
CD630_12790	3lvj	0.458438	A
CD630_35910	1cs0	0.458213	D
CD630_15360	5jfc	0.457912	S
CD630_22520	2a5h	0.457346	A
CD630_15260	1xrt	0.456057	A
CD630_34480	2fjk	0.45583	D
CD630_20310	2ord	0.455696	A
CD630_35620	3out	0.455224	A
CD630_15210	1h3e	0.455224	A
CD630_35900	1cs0	0.455056	A
CD630_24030	3ehw	0.454545	C
CD630_24260	1x9j	0.454545	H
CD630_31300	4ipn	0.454357	E
CD630_10110	2ri0	0.453815	B
CD630_21170	4zn0	0.453642	D
CD630_25840	4iqf	0.453074	C
CD630_15130	1oy0	0.450909	E
CD630_25910	1lw5	0.450581	C
CD630_08180	5okb	0.448718	D
CD630_00490	5ucm	0.446585	B
CD630_25960	1qyu	0.445545	A
CD630_27710	3gg2	0.445476	A
CD630_12120	2fl4	0.445205	A
CD630_32560	1iyw	0.44482	B
CD630_23220	4kxy	0.444444	B

CD630_08850	3cej	0.442804	A
CD630_34390	6b5f	0.442529	B
CD630_29540	3aon	0.441441	A
CD630_03940	4xkj	0.439759	B
CD630_13530	3mbh	0.43956	D
CD630_23820	3rq1	0.439141	A
CD630_25150	2zy2	0.439114	A
CD630_34210	4mlq	0.438538	A
CD630_01000	6fnp	0.436823	B
CD630_18250	4kam	0.434679	A
CD630_36640	1wst	0.434673	A
CD630_17020	4s29	0.43418	A
CD630_22700	2jg5	0.432787	A
CD630_30740	1rv8	0.432526	D
CD630_26960	4dgt	0.431818	B
CD630_01070	5wmh	0.431472	B
CD630_26110	4f1w	0.431034	B
CD630_32220	4rqo	0.43073	A
CD630_07940	2e16	0.430279	B
CD630_20140	5ym0	0.430127	A
CD630_15820	6an0	0.429929	A
CD630_26660	1ax3	0.429448	A
CD630_23320	1a3a	0.428571	D
CD630_10830	5gqs	0.428571	A
CD630_23260	5gqs	0.428571	A
CD630_21100	4xz9	0.428571	A
CD630_29561	3aon	0.428571	B
CD630_35400	4qrd	0.427907	A
CD630_08900	2q41	0.427562	C
CD630_26270	1zpv	0.426966	A
CD630_07510	4yms	0.426667	C
CD630_26790	2ztl	0.426357	D
CD630_17760	2ouk	0.426009	C
CD630_20340	1vkn	0.424419	C
CD630_34880	5i1f	0.423676	A
CD630_01540	2yaj	0.423529	D
CD630_23720	4hhe	0.421053	A
CD630_07300	3zyy	0.420561	Y
CD630_35360	4xb6	0.418283	C
CD630_19930	1vke	0.418182	D
CD630_14450	1qdl	0.417526	B
CD630_21580	5wyf	0.417234	B
CD630_17000	2hxv	0.416216	A

CD630_07970	1ydo	0.416107	B
CD630_18180	3dnf	0.415771	B
CD630_02230	4ehi	0.415686	A
CD630_34690	6n30	0.414384	G
CD630_36430	2ics	0.413793	A
CD630_26540	5jnq	0.413043	A
CD630_01870	4paw	0.412371	A
CD630_36400	3ktn	0.412121	A
CD630_09900	4nqy	0.411765	A
CD630_22060	1ad3	0.409692	B
CD630_16010	1xi3	0.409524	A
CD630_21000	5y6q	0.409396	A
CD630_10240	2d62	0.409222	A
CD630_09950	1wwk	0.408946	B
CD630_34380	1cbu	0.408602	B
CD630_04050	1vio	0.408333	A
CD630_26300	1z82	0.408163	A
CD630_21660	5fa9	0.407524	B
CD630_34600	3ooy	0.407273	A
CD630_14460	1k0g	0.40708	A
CD630_07540	2c5s	0.40665	A
CD630_34240	2zvc	0.406639	A
CD630_30840	3cwc	0.40625	A
CD630_34450	1wcr	0.405941	C
CD630_11780	1u7n	0.405882	A
CD630_12070	2o1x	0.405797	C
CD630_01370	3qnq	0.405405	A
CD630_34300	2afv	0.404762	B
CD630_24950	3w1h	0.404711	A
CD630_07530	3lvk	0.4047	A
CD630_06710	4nvs	0.403846	A
CD630_06970	4j91	0.403587	A
CD630_23560	5miq	0.403175	A
CD630_10270	4gl0	0.402857	A
CD630_18160	1q3t	0.402778	A
CD630_35430	4mzy	0.401261	A
CD630_00470	5ddt	0.400844	A
CD630_20530	2o0t	0.4	D
CD630_16500	3gfv	0.398754	A
CD630_24960	3u0o	0.39557	A
CD630_00510	6brl	0.395538	A
CD630_07200	6deb	0.393728	A
CD630_07260	2ycl	0.393407	A

CD630_34770	2hvw	0.393103	C
CD630_06960	4j7c	0.393013	I
CD630_13320	6fmt	0.390511	A
CD630_01120	3u9e	0.39	A
CD630_08840	3vue	0.389583	A
CD630_03450	5-Dec	0.388889	A
CD630_31100	3k2q	0.388614	B
CD630_07250	4djf	0.388535	F
CD630_33140	1gx7	0.388286	A
CD630_07270	1f6y	0.38806	A
CD630_30670	3bed	0.38806	A
CD630_20540	3l76	0.387909	B
CD630_21150	3j09	0.387755	A
CD630_35170	1p4a	0.386986	A
CD630_01850	5ue9	0.386957	D
CD630_14520	3qbc	0.386905	A
CD630_34590	4kxw	0.386581	A
CD630_29590	3aou	0.386503	E
CD630_21640	3pqf	0.385093	C
CD630_16990	1kzl	0.384615	A
CD630_15950	4n69	0.382653	B
CD630_25670	2r48	0.382353	A
CD630_26640	4bub	0.382231	A
CD630_23710	5kxj	0.381062	A
CD630_29860	6fmt	0.380282	A
CD630_10600	2ist	0.38	A
CD630_01360	2wy2	0.37963	D
CD630_08610	2l2q	0.37963	A
CD630_10100	2vhl	0.37931	B
CD630_32290	4f3y	0.378486	A
CD630_35270	1q12	0.377644	B
CD630_14890	3dhw	0.376947	C
CD630_23420	5j78	0.37581	A
CD630_04920	1nrz	0.375796	B
CD630_08820	5l6v	0.375648	D
CD630_25030	5xaa	0.375427	A
CD630_34510	2q5r	0.375405	D
CD630_02990	1vm7	0.375	A
CD630_25120	2f3g	0.375	B
CD630_32730	2k5l	0.375	A
CD630_35920	3ldv	0.37395	B
CD630_32820	4mtj	0.373891	B
CD630_32260	6bdx	0.373494	A

CD630_19140	5ysn	0.372014	D
CD630_18350	1umf	0.370474	C
CD630_01160	1yd7	0.370474	A
CD630_32530	1o5z	0.37037	A
CD630_32610	2ouk	0.370079	D
CD630_27370	1j31	0.369403	D
CD630_30680	2jzo	0.36875	D
CD630_26230	3cpg	0.367089	A
CD630_21550	3iiz	0.365651	A
CD630_35180	1gqy	0.364444	B
CD630_02870	3lfj	0.364198	B
CD630_04890	1kut	0.362832	A
CD630_27440	5iqr	0.360544	8
CD630_09940	2dr1	0.360335	B
CD630_31350	1rv8	0.359589	D
CD630_23210	3ooy	0.359477	A
CD630_19450	4nvs	0.359477	A
CD630_12260	4iiw	0.359331	A
CD630_27790	2x5s	0.358543	B
CD630_25940	3qe7	0.35732	A
CD630_34270	300000	0.356021	E
CD630_22360	5nck	0.355932	A
CD630_21730	1ysj	0.355499	A
CD630_14510	5far	0.355372	C
CD630_25900	3ekm	0.355072	E
CD630_16000	3hpd	0.354962	A
CD630_15910	6hra	0.35461	A
CD630_15520	4wd0	0.354167	A
CD630_00360	6cfo	0.354037	A
CD630_13220	3ab4	0.353808	E
CD630_35300	3puz	0.353474	B
CD630_20500	4lgt	0.352941	A
CD630_28830	3qnq	0.352403	A
CD630_00450	6btd	0.351852	A
CD630_22000	3ffh	0.351499	A
CD630_30480	3qnq	0.350446	A
CD630_21190	5wat	0.350168	A
CD630_34670	6n2z	0.348837	H
CD630_04910	5im4	0.348148	I
CD630_20270	3n5f	0.348148	A
CD630_16820	3fz0	0.347826	C
CD630_31950	2vf8	0.347395	B
CD630_10040	2isb	0.346154	A

CD630_07080	2qyv	0.345756	A
CD630_04900	4uek	0.345714	A
CD630_34320	3ftb	0.345506	A
CD630_34400	3dcd	0.344828	B
CD630_20870	1rm6	0.344173	A
CD630_31030	1ax3	0.343558	A
CD630_08330	1amj	0.343214	A
CD630_21180	4f4f	0.342799	B
CD630_36700	5b89	0.342105	A
CD630_22450	1x55	0.341935	A
CD630_24840	3l8a	0.341837	A
CD630_11340	4mtq	0.341463	B
CD630_16280	4ecl	0.341292	D
CD630_16140	4yer	0.340909	A
CD630_25240	5das	0.340611	D
CD630_24250	3uf6	0.340067	A
CD630_00420	1tvm	0.34	A
CD630_07170	3kjg	0.339768	B
CD630_30510	3cqy	0.339426	B
CD630_18330	3okf	0.338983	A
CD630_33080	1oyr	0.33853	C
CD630_27420	4ysb	0.338235	A
CD630_23230	4ej6	0.338192	A
CD630_34730	2wgm	0.337209	R
CD630_12200	5c7q	0.337079	A
CD630_01020	4huq	0.337079	T
CD630_15930	6hrb	0.336538	C
CD630_27620	1f75	0.336449	A
CD630_09270	3lx6	0.336134	B
CD630_15590	5jen	0.33564	A
CD630_36270	3a5f	0.335548	B
CD630_21990	1yd7	0.335244	A
CD630_22770	6btd	0.334884	A
CD630_26530	3lk7	0.334812	A
CD630_31060	3r8r	0.333333	K
CD630_30820	4mge	0.333333	A
CD630_19390	4hr7	0.333333	B
CD630_01030	1vs3	0.333333	B
CD630_28810	2i5i	0.333333	B
CD630_34440	2l2q	0.333333	A
CD630_32790	2jzo	0.333333	D
CD630_02850	3lfj	0.333333	B
CD630_25160	3nxk	0.332326	G

CD630_23610	2it1	0.332	B
CD630_18060	3lki	0.331269	B
CD630_28750	5b57	0.330769	D
CD630_07150	3tng	0.330033	A
CD630_22130	1g5c	0.329787	C
CD630_26920	3ftb	0.329609	E
CD630_13870	3va7	0.329268	A
CD630_20330	1vz6	0.328395	A
CD630_09000	1vci	0.328042	A
CD630_03340	3my7	0.327273	C
CD630_10490	5dhq	0.327068	C
CD630_23240	4ueo	0.325714	A
CD630_11290	2if2	0.325	C
CD630_18340	3nvs	0.324943	A
CD630_20990	1rm6	0.324934	A
CD630_20730	1rm6	0.324219	A
CD630_11660	4pg5	0.32419	A
CD630_25140	2gn1	0.323457	B
CD630_15510	3zr4	0.323383	F
CD630_17470	6n91	0.322188	A
CD630_18380	2pt5	0.322034	A
CD630_10760	2jzh	0.320513	A
CD630_13150	1t6x	0.320388	B
CD630_24360	2yvx	0.320346	B
CD630_17750	4ymv	0.320197	C
CD630_01210	3ipo	0.320186	B
CD630_28350	4r1i	0.32	A
CD630_30000	2v9d	0.319865	C
CD630_31820	1v51	0.318786	A
CD630_03000	5hsg	0.31875	A
CD630_03270	3gfo	0.318681	A
CD630_15490	3cq5	0.318052	A
CD630_29660	3my7	0.317186	D
CD630_31770	4us8	0.31653	A
CD630_10740	5t3u	0.316176	B
CD630_15800	4pg7	0.316049	B
CD630_35510	2x3l	0.315565	A
CD630_34530	2vhl	0.315104	A
CD630_19780	5xu1	0.314286	A
CD630_07370	3dcp	0.312741	A
CD630_35330	4yms	0.312236	A
CD630_14900	3tuj	0.311927	B
CD630_10690	4gc3	0.310606	A

CD630_08320	3rmj	0.309824	B
CD630_26550	4cvl	0.308534	A
CD630_18200	3t81	0.30853	B
CD630_30800	3k1s	0.307692	H
CD630_02840	3lfh	0.30597	A
CD630_00380	3duf	0.304598	I
CD630_33570	4e08	0.304124	A
CD630_23190	5umf	0.303167	B
CD630_13000	3h8f	0.302277	E
CD630_22390	5nv9	0.302	A
CD630_03130	4umw	0.301887	A
CD630_01740	6elq	0.301738	B
CD630_10010	5xu1	0.301724	A
CD630_35580	1bia	0.301538	A
CD630_02060	1a6j	0.3	A

49. Kavvas, E. S. et al. Updated and standardized genome-scale reconstruction of *Mycobacterium tuberculosis* H37Rv, iEK1011, simulates flux states indicative of physiological conditions. *BMC Syst. Biol.* **12**, 25 (2018).

50. Norsigian, C. J., Kavvas, E., Seif, Y., Palsson, B. O. & Monk, J. M. iCN718, an updated and improved genome-scale metabolic network reconstruction of *Acinetobacter baumannii* AYE. *Front. Genet.* **9**, 121 (2018).

51. Monk, J. M. et al. iML1515, a knowledgebase that computes *Escherichia coli* traits. *Nat. Biotechnol.* **35**, 904–908 (2017).

52. Nogales, J., Palsson, B. Ø. & Thiele, I. A genome-scale metabolic reconstruction of *Pseudomonas putida* KT2440: i JN746 as a cell factory. *BMC Syst. Biol.* **2**, 79 (2008).

53. Oh, Y.-K., Palsson, B. O., Park, S. M., Schilling, C. H. & Mahadevan, R. Genome-scale reconstruction of metabolic network in *Bacillus subtilis* based on high-throughput phenotyping and gene essentiality data. *J. Biol. Chem.* **282**, 28791–28799 (2007).

54. Nagarajan, H. et al. Characterizing acetogenic metabolism using a genome-scale metabolic reconstruction of Clostridium ljungdahlii. *Microb. Cell Fact.* **12**, 118 (2013).

55. Ebrahim, A. et al. Do genome-scale models need exact solvers or clearer standards? *Mol. Syst. Biol*. **11,** 10 (2015).

56. Lieven, C. et al. MEMOTE for standardized genome-scale metabolic model testing. *Nat. Biotechnol.* 1–5 (2020).

57. Monot, M. et al. Reannotation of the genome sequence of *Clostridium difficile* strain 630. *J. Med. Microbiol.* **60**, 1193–1199 (2011).

58. Ferreyra, J. A. et al. Gut microbiota-produced succinate promotes *C. difficile* infection after antibiotic treatment or motility disturbance. *Cell Host Microbe* **16**, 770–777 (2014).
